# Metabolomics Analysis of Mesenchymal Stem Cell (MSC) Therapy in a Phase I Clinical Trial of Septic Shock: An Exploratory Study

**DOI:** 10.3390/metabo13111142

**Published:** 2023-11-10

**Authors:** Mohammad M. Banoei, Lauralyn A. McIntyre, Duncan J. Stewart, Shirley H. J. Mei, David Courtman, Irene Watpool, John Granton, John Marshall, Claudia dos Santos, Keith R. Walley, Kenny Schlosser, Dean A. Fergusson, Brent W. Winston, on behalf of Canadian Critical Care Trials Group (CCCTG), on behalf of Canadian Critical Care Translational Biology Group (CCCTBG)

**Affiliations:** 1 Department of Critical Care Medicine, Cumming School of Medicine, University of Calgary, Calgary, AB T2N 4Z6, Canada; mmbanoei@ucalgary.ca; 2 Department of Medicine (Division of Critical Care), University of Ottawa, Ottawa, ON K1H 8L6, Canada; lmcintyre@ohri.ca; 3 Ottawa Hospital Research Institute, Ottawa, ON K1H 8M5, Canada; duncan.stewart@ohri.ca (D.J.S.); smei@ohri.ca (S.H.J.M.); dcourtman@ohri.ca (D.C.); irene.watpool@ohri.ca (I.W.); kenny.schlosser@ohri.ca (K.S.); dafergusson@ohri.ca (D.A.F.); 4 Department of Epidemiology and Community Medicine, University of Ottawa， Ottawa, ON K1H 8L6, Canada; 5 Department of Cell and Molecular Medicine, University of Ottawa, Ottawa, ON K1H 8M5, Canada; 6 Department of Regenerative Medicine, Ottawa Hospital Research Institute, Ottawa, ON K1H 8M5, Canada; 7 Department of Medicine, University of Toronto, Toronto, ON M5G 2N2, Canada; john.granton@uhn.ca; 8 Department of Surgery and Critical Care Medicine, Keenan Research Centre of the Li Ka Shing Knowledge Institute, St. Michael’s Hospital, The University of Toronto, Toronto, ON M5B 1W8, Canada; john.marshall@unityhealth.to (J.M.); dossantosc@smh.ca (C.d.S.); 9 Centre for Heart Lung Innovation, University of British Columbia, Vancouver, BC V6Z 1Y6, Canada; keith.walley@hli.ubc.ca; 10 Departments of Medicine and Biochemistry and Molecular Biology, Cumming School of Medicine, University of Calgary, Calgary, AB T2N 4Z6, Canada

**Keywords:** biomarker, mesenchymal stem cells, septic shock, clinical trial, metabolomics, cell therapy

## Abstract

Sepsis is the result of an uncontrolled host inflammatory response to infection that may lead to septic shock with multiorgan failure and a high mortality rate. There is an urgent need to improve early diagnosis and to find markers identifying those who will develop septic shock and certainly a need to develop targeted treatments to prevent septic shock and its high mortality. Herein, we explore metabolic alterations due to mesenchymal stromal cell (MSC) treatment of septic shock. The clinical findings for this study were already reported; MSC therapy was well-tolerated and safe in patients in this phase I clinical trial. In this exploratory metabolomics study, 9 out of 30 patients received an escalating dose of MSC treatment, while 21 patients were without MSC treatment. Serum metabolomics profiling was performed to detect and characterize metabolite changes due to MSC treatment and to help determine the sample size needed for a phase II clinical trial and to define a metabolomic response to MSC treatment. Serum metabolites were measured using ^1^H-NMR and HILIC-MS at times 0, 24 and 72 h after MSC infusion. The results demonstrated the significant impact of MSC treatment on serum metabolic changes in a dose- and time-dependent manner compared to non-MSC-treated septic shock patients. This study suggests that plasma metabolomics can be used to assess the response to MSC therapy and that treatment-related metabolomics effects can be used to help determine the sample size needed in a phase II trial. As this study was not powered to detect outcome, how the treatment-induced metabolomic changes described in this study of MSC-treated septic shock patients are related to outcomes of septic shock in the short and long term will need to be explored in a larger adequately powered phase II clinical trial.

## 1. Introduction

Septic shock is associated with a severely dysregulated host immune response to an initial infection following perturbations in physiological functions such as cardiovascular, respiratory, metabolic, hormonal, inflammatory, and innate and adaptive immunity [1,2]. Establishing metabolite-based biomarkers in patients can help define specific metabolic profiles that may indicate the presence of certain diseases or can be used to track pharmacologic responses to therapies [3]. Recently, MSC therapy for septic shock was evaluated for the first time in a phase I dose escalation trial (Cellular Immunotherapy for Septic Shock: CISS) [4]. The study showed that MSC therapy was a well-tolerated and safe treatment for septic shock patients [4]. The plasma level of cytokines (i.e., IL-6, IL-8, IL-1β and IL-10) and protein markers (i.e., ANGPT2, RAGE and PSD) in MSC-treated patients with septic shock were found not well correlated with MSC treatment [4,5,6,7], strongly suggesting that they will not effectively predict treatment effects of MSC therapy. They also did not reflect any adverse events, as there were no adverse events associated with MSC treatment in this phase I trial other than fever [4].

We aimed to explore the impact of MSC treatment of septic shock patients on serum metabolites. Notably, no significant changes were found in the clinical phenotypes [4] and blood inflammatory markers [7] up to 72 h after MSC infusion in the phase I clinical trial patients. We sought to explore if and how metabolites may change with respect to the time and dose of MSCs used up to 72 h post-MSC treatment. We also questioned whether this study would allow us to estimate the sample size needed for a phase II study of MSC therapy for septic shock. Finally, we were interested in investigating whether metabolomics would help us understand the potential underlying mechanisms involved in MSC treatment of septic shock. In this metabolomics substudy of the phase I clinical trial of MSC treatment in septic shock, we investigated serum metabolite alterations in patients undergoing MSC treatment for septic shock using proton nuclear magnetic resonance (^1^H-NMR) spectroscopy and hydrophilic interaction liquid chromatography–mass spectrometry (HILIC-MS), measuring an extensive panel of over 150 plasma metabolites. We compared the metabolites in the serum of MSC-treated septic shock patients vs. non-MSC-treated septic shock controls at times 0, 24 and 72 h. We also examined changes in serum metabolites in MSC-treated septic shock patients from baseline to 72 h post-treatment.

## 2. Materials and Methods

### 2.1. Patients and Controls Enrollment

The MSC treatment of septic shock trial was approved by Health Canada, the Ottawa Health Sciences Network Research Ethics Board (OHSN-REB #: 20140809-011) and the Conjoint Health Research Ethics Board of the University of Calgary, Canada. Details about the phase I study have been previously described, including rationale, patient enrollment, inclusion and exclusion criteria, the preparation of bone-marrow-derived MSCs and clinical outcomes [4]. The observational cohort included 21 patients with septic shock who did not receive MSC treatment. Nine patients with septic shock were enrolled in the interventional cohort and were further subcategorized into three subgroups that received an escalating dose of 0.3, 1.0 and 3.0 million MSCs (cells) per kg with a maximum of 300 million cells per patient. The groups were called low- (*n* = 3), medium- (*n* = 3) and high- (*n* = 3) dose groups, respectively. Serum samples were taken at times 0, 24 and 72 h after MSC infusion (or noninfusion in the control group) using a standard operating procedure (SOP); serum was isolated and frozen at −80 °C until it was used for metabolomics analysis.

### 2.2. Metabolite Identification and Profiling

For ^1^H-NMR analysis, patients’ serum was filtered using 3 kDa NanoSep microcentrifuge filters, and ^1^H-NMR spectroscopy was performed using a 600 MHz NMR spectrometer (Bruker BioSpin Ltd., Milton, ON, Canada), as previously described [8]. NMR spectra were processed and profiled in a nontargeted approach using ChenomX NMR Suite 7.1 (ChenomX Inc., Edmonton, AB, Canada) [9,10]. Fifty-six metabolites were quantified via ^1^H-NMR, including sugar alcohols, sugars, amino acids and volatile compounds.

For HILIC-MS, metabolites were extracted from serum using 50% methanol solvent followed by centrifugation and transferring supernatant to the mass spectrometer. A UHPLC-MS (Q Exactive HF Hybrid Quadrupole-Orbitrap Mass Spectrometer, Thermo-Fisher, Calgary, AB, Canada) was used to analyze the serum samples. We used an ultrahigh-performance liquid chromatography system using a 2.1 mm × 100 mm long Syncronis HILIC (Thermo-Fisher, Calgary, AB, Canada) LC column that was packed in-house with 3 µm pore Hypercarb particles. More details about HILIC-MS analysis can be found in the Supplementary Materials. Maven software was used to process HILIC-MS spectral data for the identification and quantification of metabolites [11,12]. Using an untargeted approach, 133 metabolites were quantified using HILIC-MS and consisted of amino acids, organic acids, sugars, sugar alcohols and acylcarnitines compounds.

### 2.3. Data Analysis

Both univariate and multivariate data analyses were applied to extract information from the metabolomic datasets. Univariate analyses were used as complementary methods to multivariate analysis to provide more information on metabolomic profiles as well as to provide information on each metabolite individually. Principal component analysis (PCA) was performed using datasets derived from serum samples to evaluate metabolite inter-relationships and aggregation of cohorts using the metabolomics dataset between MSC-treated and non-MSC-treated control cohorts. Partial least square regression (PLSR) was applied to show the relation of the most important metabolites obtained via OPLS-DA and PLS-DA analysis to the separation of the cohorts. MetaboAnalyst [13] and MetaBox [14] were used for univariate data analyses. SIMCA-P v.15.0.2 (Umetrics AB, Umeå, Sweden) and MetaboAnalyst 5.0 software (Mcgill University, Montreal, QC, Canada) were used for multivariate analyses. Parallel pathway analyses were performed using MetaboAnalyst 4.0 (Mcgill University, Montreal, QC, Canada) [15], a free web-based tool, and Cytoscape 3.6.0 (Mcgill University, Montreal, QC, Canada) [16]. For more details, see the Supplementary Materials.

## 3. Results

### 3.1. Patient Cohorts

We enrolled 9 MSC-treated patients with septic shock and 21 non-MSC-treated septic shock controls. Age, sex and APACHE II were not significantly different (*p* > 0.05) between MSC-treated and control cohorts. For a detailed explanation of patient characteristics, see McIntyre et al., 2018 [4]. The mean age was 68.6 ± 19.0 and 57.8 ± 16.6, and the APACHE II mean was 22.2 ± 4.7 and 24.05 ± 4.3 for the interventional and observational cohorts, respectively (both *p* > 0.05). Ag—and sex-matched MSC-treated patients (*n* = 9) and untreated patients (*n* = 9) were used to observe the effect of MSC treatment on metabolic profiles. Patients’ characteristics, clinical outcomes and adverse events have previously been shown by McIntyre et al. (2018) [4]. Although elevated temperature was noted in the MSC-treated group, no significant difference was observed in temperature between the MSC-treated and nontreated control groups at the beginning and end of MSC infusion. Also, the nontreated control cohort had no significant deterioration in heart rate, mean arterial pressure, PaO_2_/FiO_2_ ratio and oxygenation index in the first 72 h and in multiple organ dysfunction (MOD) scores for the first 7 days (see McIntyre et al. (2018) [4]).

### 3.2. Unveiling the Impact of MSC Treatment on Metabolites Biopattern Using an Unsupervised Method

PCA depicted the effect of MSC treatment on metabolite profile, showing relative clustering of the MSC-treated cohort and the nontreated controls (samples at 24 h and 72 h) that was greater using the HILIC-MS dataset (Figure S1) compared to the ^1^H-NMR dataset (Figure S1A,B, see Supplementary Materials). Specifically, the effect of MSC treatment was observed via the relative clustering of two cohorts at two time points 24 h and 72 h, separately, using the HILIC-MS dataset (Figure S2A–D). This grouping is also seen when comparing MSC-treated and all nontreated controls (*n* = 21) for the samples at 24 and 72 h (Figure S3A,B). The results obtained via PCA presented 60% metabolomics profile variability (R^2^X = 0.602) across MSC-treated and nontreated control cohorts, showing a high level of certainty for MSCs’ impact on metabolites biopattern. In addition, PCA shows no grouping or clustering among baseline (T0) samples of MSC-treated (*n* = 9) and non-MSC-treated age- and sex-matched controls (*n* = 9) (Figure S4A–B). PCA also revealed no grouping or clustering of baseline (T0) samples between the treated baseline (*n* = 9) and all nontreated controls (*n* = 21) (Figure S5A,B), showing no significant change in metabolite composition in the absence of MSC therapy.

### 3.3. MSC Treatment Caused a Significant Metabolic Alteration over 24 and 72 h

**Figure 1:**
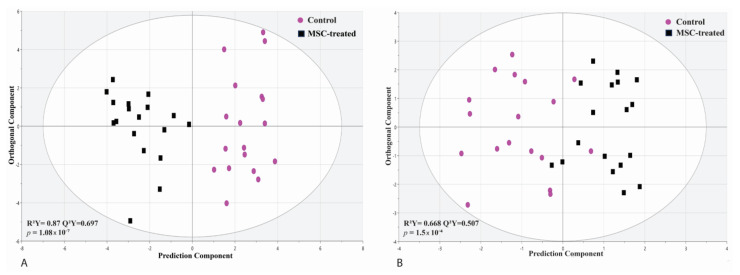
(**A**) OPLS-DA analysis on the HILIC-MS dataset shows a robust separation between MSC-treated septic shock patient samples (*n* = 9) and non-MSCtreated septic shock patients (*n* = 9) using 42 metabolites (age- and sex-matched samples at 24 and 72 h). R^2^X = 0.939 reflects a model with a good fit of the data. The Q^2^Y = 0.737 and *p*-value (*p* = 3.95 × 10^−6^) indicate a significant predictive model. (**B**) OPLS-DA analysis of the ^1^H-NMR dataset shows a good separation between MSC-treated septic shock patient samples (*n* = 9) and non-MSC-treated septic shock patients (*n* = 9) using 17 metabolites (age- and sex-matched samples at 24 and 72 h). R^2^X = 0.668 reflects a model with a good fit of the data. The Q^2^Y (0.507) and *p*-value (*p* = 0.00015) indicate a significant predictive model. The X-axis and Y-axis present the prediction orthogonal components, respectively.

The serum-based metabolic biosignature was significantly altered after MSC infusion in septic shock patients compared to nontreated control patients using samples at 24 and 72 h postinfusion. Metabolomics data generated from ^1^H-NMR and HILIC-MS analysis were informative in showing the impact of MSCs on metabolic changes. In the HILIC-MS dataset, this MSC impact was highly predictive (Q^2^ = 0.697) and more significant (*p* = 1.08 × 10^−7^) compared to the ^1^H-NMR dataset Q^2^ = 0.507 and *p* = 0.00015 using OPLS-DA models (Figure 1A,B). These predictive models were obtained based on 47 and 17 of the most differentiating metabolites (VIP > 1.0) between the MSC-treated cohort and the nontreated septic shock controls using HILIC-MS and ^1^H-NMR, respectively. These differentiating metabolites are represented in the coefficient plots (Figures S6 and S7).

Samples at baseline (T = 0) were not metabolically different prior to MSC treatment for each of the ^1^H-NMR and HILIC-MS datasets. The metabolomic profile of baseline samples (T = 0) was further examined using 47 (HILIC-MS) and 17 (NMR) metabolites which contributed to the dichotomy of MSC-treated and nontreated cohorts at 24 and 72 h. Table 1 summarizes the OPLS-DA model characteristics for the discrimination of samples at baseline and post-MSC treatment, indicating a lack of significant metabolic changes at baseline (T = 0), whereas there were highly predictive and significant changes in metabolite biosignatures following MSCs therapy at 24 and 72 h. The validity of R^2^ and Q^2^ values was confirmed via permutation testing with 200 repetitions (Figure S13D).

**Figure 2:**
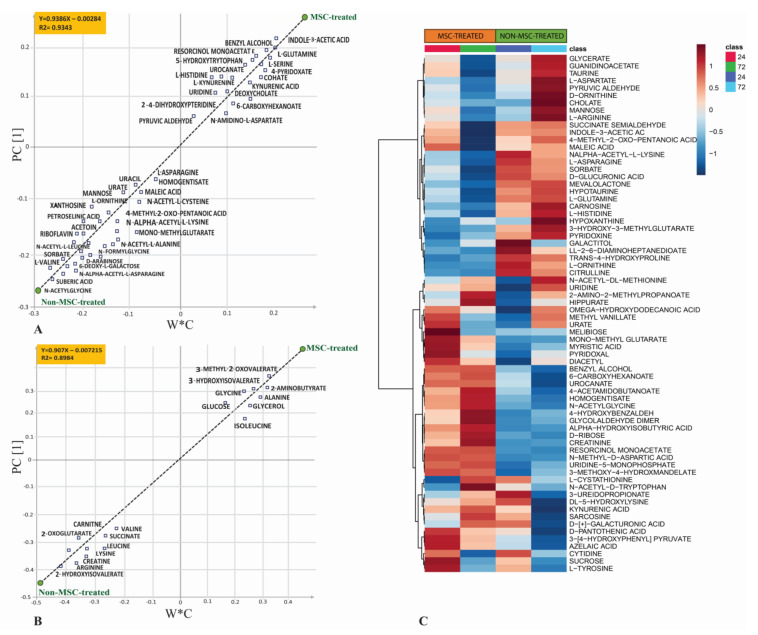
PLS regression shows a strong relationship R^2^ = 0.93 and R^2^ = 0.89 between the most important metabolites and separation of MSC-treated septic shock patients from non-MSC-treated septic shock patients using the (**A**) HILIC-MS dataset and (**B**) ^1^H-NMR datasets. W*C (X-axis) is the weight of the variables in the correlation between X (metabolites) and Y (treatment group). PC1 (Y-axis) presents the principal component 1. Heatmap of the average of top 60 metabolites between MSC-treated and non-MSC-treated groups at two time points (24 and 72 h) from the HILIC-MS dataset. (**C**) Heatmap shows the average metrabolite concentration between MSC-treated and control based on the sampling times at 24 and 72h.

In the HILIC-MS dataset, *t*-test analysis showed that 38 metabolites were significantly different (*p* < 0.05) between the two cohorts after MSC infusion, including 22 metabolites with significant FDR differences (q < 0.05) (Table S1), while there were no significant differences in metabolites at baseline between the two cohorts (Table 1). In the NMR dataset, eight metabolites showed significant differences (*p* < 0.05) in metabolite concentrations between MSC-treated and nontreated cohorts, but none showed significant FDR (<0.05) changes (Table S2). PLS regression analysis revealed a strong relationship between the most differentiating metabolites and the separation of the MSC-treated from nontreated septic shock cohorts. PLSR demonstrated R^2^ = 0.93 and R^2^ = 0.89 for HILIC-MS and ^1^H-NMR datasets, respectively (Figure 2A,B). Heatmap analysis (Figure 2C) shows the average concentrations of the top 70 metabolites between two MSC and non-MSC-treated groups at 24 and 72 h post-treatment. 

OPLS-DA analysis showed a very predictive model (Q^2^Y = 0.729, *p* = 2.7 × 10^−9^) to separate the MCS-treated patients from all non-MSC-treated patients using the HILIC-MS dataset (Figure S8).

The data suggest that the metabolite alterations due to MSC treatment were better reflected by the HILIC-MS dataset, showing distinct metabolic biosignatures for the MSC-treated cohort from nontreated controls compared to the NMR dataset (for further explanation of the HILIC-MS and NMR analysis, see Supplementary Materials).

### 3.4. MSC Treatment Exhibited More Pronounced Metabolite Changes at 72 h Compared to 24 h

In the HILIC-MS dataset, the metabolite profiles shifted to a greater degree within 72 h, when compared to 24 h, in MSC-treated septic shock patients compared to non-MSC-treated controls. Results showed that 48 out of 133 metabolites significantly (*p* < 0.05) changed between the MSC-treated and control cohorts at 72 h, including 21 metabolites with significant FDR (q < 0.05) differences (Table S3). However, only six metabolites significantly changed (*p* < 0.05) within 24 h between MSC-treated and nontreated patients. Multivariate models also revealed metabolite changes at the individual 24 and 72 h of treated vs. nontreated cohorts, with a greater difference after 72 h. Also, metabolomics differences were greater at 72 h than at 24 h between the MSC-treated septic shock and nontreated septic shock control patients for ^1^H-NMR analysis using MVA (Figure S9A,B) and UVA analyses. HILIC-MS results show that marked time-dependent metabolite concentration changes in the serum with several altered metabolites significantly increased from 24 to 72 h due to MSC infusion (Figure S14).

### 3.5. Metabolite Alterations Were Significantly Correlated with the Dose of MSCs

**Figure 3:**
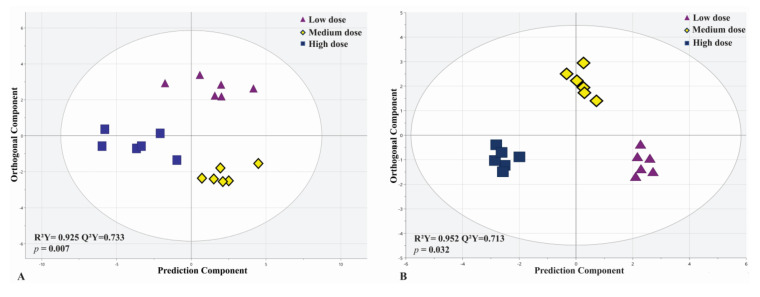
PLS-DA analysis shows distinct separation among three MSC-treated dosage subgroups (low-, medium- and high-dose) with strong predictability (Q^2^Y = 0.864, Q^2^Y = 0.713) and significant models (*p* = 0.009 and *p* = 0.032) for both HILIC-MS (**A**) and ^1^H-NMR (**B**) datasets.

**Figure 4:**
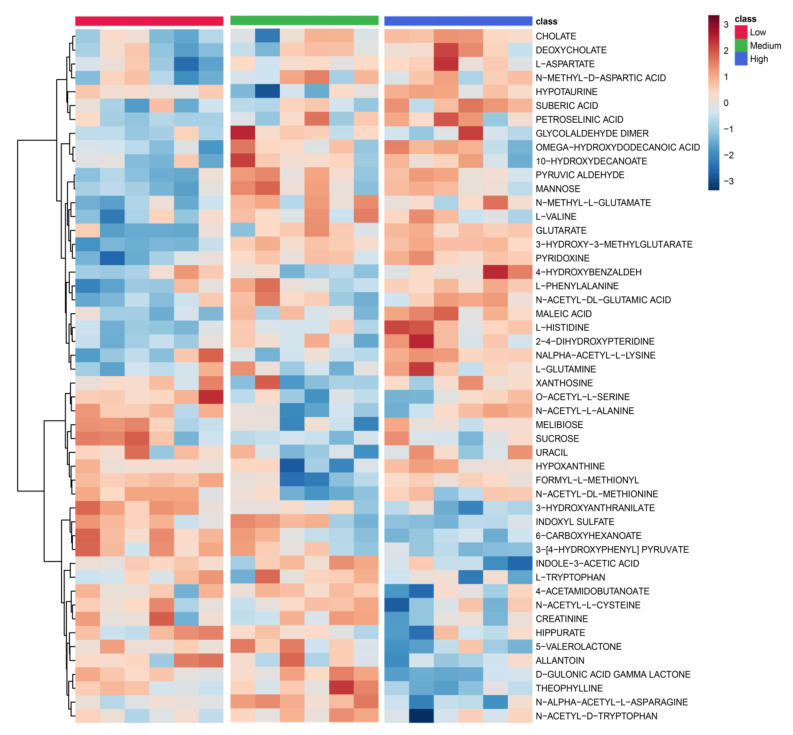
Heatmap shows metabolite profiling differences in the HILIC-MS dataset between the three different dose groups (low, medium and high) of MSC-treated septic shock patients and the metabolite-based heterogeneity among each group.

We further investigated the serum metabolomic profiles of septic shock patients treated with low, medium and high doses of MSCs. Differences in metabolic profiles were observed among the three dosage groups (low, medium and high doses of MSCs) via aggregation of samples (Figure S10A,B) in an unsupervised PCA approach. Further analysis showed a pattern of change in metabolite profiles from low to high dose (left to right) based on a pool of 133 metabolites (Figure S10A). Despite the small numbers of patients in each dose treatment group (*n* = 3) at each time point (24 h and 72 h), both MVA and UVA approaches demonstrated that low-, medium- and high-dose subgroups can differentially change the metabolic biosignature in the MSC-treated cohort. Three subgroups can be separated with a highly predictive (Q^2^Y = 0.733) and a statistically significant PLS-DA model (*p* = 0.009) using a metabolic profile including 55 of the most differentiating metabolites from the HILIC-MS dataset (Figure 3). To prevent overfitting of the data, the validity of R^2^ and Q^2^ values were confirmed using permutation testing with 200 repetitions (Figure S13C). A heatmap plot depicts the metabolomic differences between the three-dose subgroups (Figure 4) in top metabolites for different doses of MSC treatment. ANOVA analysis also revealed that 43 metabolites statistically differed between the three dose groups at 24 h and 72 h, including 21 metabolites with significant FDR differences (q < 0.05) (Table S4). This table shows that all metabolites (21 metabolites with an FDR < 0.05) significantly changed between any combination of the two groups, supporting the role of MSC dose dependence in the alteration of the patient’s metabolites. It was observed that the high-dose group exerted the greatest change in metabolites followed by medium and low doses using *t*-test analysis. The ^1^H-NMR dataset provided similar results in the separation of the different-dose subgroups but was less predictive and significant than the HILIC-MS analysis (Figure 3B).

### 3.6. Serum Metabolite Profile Significantly Changed in 24 and 72 h following MSC Treatment vs. Baseline within Group Analysis

**Figure 5:**
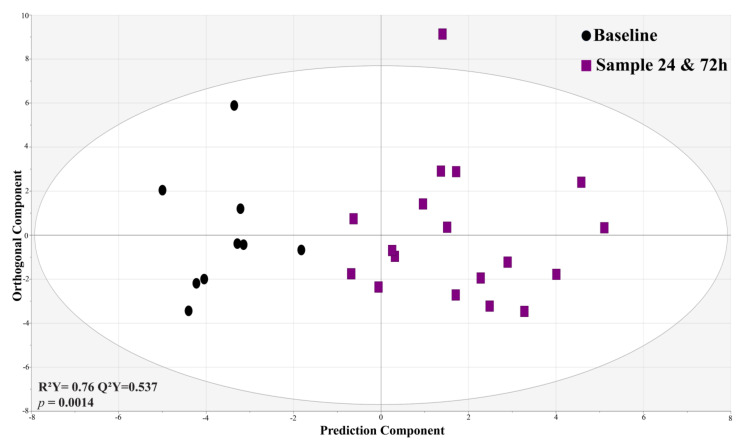
OPLS-DA shows a predictive (Q^2^Y = 0.537) and significant separation in patient serum metabolites (48) from baseline (T0) compared to serum metabolites after MSC infusion (combined at 24 and 72 h). This compares metabolites from the MSC-treated cohort at 24 and 72 h vs. the metabolites at baseline (T0) prior to treatment.

We investigated the metabolite alterations within each MSC-treated and nontreated cohort to understand how metabolomic profiles change over time after receiving MSC treatment compared to metabolites at baseline. HILIC-MS data showed a significant (*p* = 0.0014) and predictable (Q^2^Y = 0.537) metabolic alteration between baseline samples and samples at 24 and 72 h in the MSC-treated cohort (Figure 5). The validity of R^2^ and Q^2^ values was confirmed using permutation testing with 200 repetitions (Figure S13A–D).

We found that the metabolite changes are higher at 72 h than at 24 h with respect to baseline samples in the MSC-treated cohort for both the HILIC-MS and ^1^H-NMR datasets. Metabolite changes for 72 h are greater than for 24 h compared to baseline, as 72 h samples stay farther from baseline for both analytical platforms (Figure S11A,B). Importantly, in the nontreated control cohort, we observed that metabolites from samples at 72 h significantly changed compared to baseline (T = 0) for both ^1^H-NMR and HILIC-MS datasets, but not as dramatically as the MSC-treated group. That is, not surprisingly, metabolites do change with septic shock over time (these patients were treated conventionally with mechanical ventilation, antibiotics, fluid and vasopressor agents). There are alterations in biochemical pathways that are impacted for numerous reasons in septic shock, such as the pathological processes of septic shock and/or the effects of current clinically relevant treatments. The metabolite alterations were also significant between samples at 24 h and 72 h for the MSC-treated cohort (Figure S12). Thus, significant metabolite changes were seen in the MSC-treated cohort between baseline vs. 24 h, baseline vs. 72 h and 24 h vs. 72 h post-treatment with MSC. These changes were more evident in the HILIC-MS dataset than in the NMR dataset. The current results demonstrate that MSC treatment yields a significant alteration in metabolites compared to the baseline and that metabolites differ over 24 h and 72 h post-MSC treatment.

### 3.7. Characterization of the Key Metabolic Pathways

Table S6 summarizes the most important involved biochemical pathways obtained via Cytoscape and MetaboAnalyst between the MSC-treated septic shock cohort and nontreated controls. Using a multivariate analysis approach, we demonstrated that the tryptophan–kynurenine pathway is upregulated in MSC-treated patients with septic shock. Figure S6 shows the increase in multiple metabolites such as L-kynurenine, 3-hydroxy kynurenine, 5-hydroxy-L-tryptophan, kynurenic acid and N-acetyl-L-tryptophan, which contribute to the tryptophan–kynurenine pathway or kynurenine metabolism. Current evidence builds positive connections between the tryptophan–kynurenine pathways with immunoregulatory mechanisms in numerous pathological conditions [17,18]. Our results show a decrease in cholate and deoxycholate, two bile acid products that can be correlated with the induction of the inflammatory response [19]. Additionally, we show an increase in 4-pyridoxate (vitamin B6) and L-histidine in MSC-treated patients, which can be associated with the initiation of inflammation [20,21]. We also found sufficient evidence for the downregulation of glycine metabolism in the MSC-treated cohort. In addition, we observed a decrease in metabolites such as N-formylglycine, N-acetylglycine, sarcosine (N-methylglycine), threonine, serine and cystathionine (Figure S6), which are central to glycine metabolism. Glycine metabolism could be associated with anti-inflammatory and immunomodulatory mechanisms [22] that are suppressed after MSC treatment in patients with septic shock. Moreover, reduced concentrations were observed for N-acetyl-L-lysine, DL-5-hydroxylysine and N-alpha-acetyl-L-lysine after MSC treatment, indicating the downregulation of lysine metabolism. Lysine metabolism can be involved in the anti-inflammatory mechanisms of inflammatory cells like macrophages and dendritic cells [23] that are reduced in MSC-treated patients. Table S6 summarizes the most important biochemical pathways obtained via Cytoscape and MetaboAnalyst between the MSC-treated septic shock cohort and non-MSC-treated controls.

## 4. Discussion

It has already been shown that MSC treatment is a well-tolerated and safe therapy for patients with septic shock [4]. It has also been shown that there are no significant changes in inflammatory biomarkers [7] within 72 h post-MSC treatment. We now importantly show that MSC treatment significantly alters the metabolomics profile of patients with septic shock compared to nontreated septic shock control patients at 24 and 72 h post-treatment. These changes in metabolites should allow one to monitor MSC treatment of septic shock, which is not possible if one just looks at inflammatory biomarkers/cytokines. We also revealed a significant impact of MSC therapy on metabolite biopatterns in MSC-treated patients over time from 24 h to 72 h, i.e., there is a time-dependent change in metabolites post-MSC treatment in septic shock over 72 h. Moreover, we also showed dose-dependent metabolite alterations in patients treated with low, medium and high doses of MSCs over 72 h post-treatment. Specifically, the high-dose cohort showed greater changes in metabolites compared to medium- and low-dose treatment when compared to the baseline (T0). Finally, with caution in the interpretation of the results because of the small number of patients, we were able to examine metabolic pathways that appear to be affected by MSC therapy of septic shock patients, and these are highlighted below.

This study illustrated the opportunities for real-time monitoring of metabolite alterations in a time-dependent and dose-dependent fashion in sepsis and septic shock investigation and treatment with MSCs [24]. This may ultimately lead to the identification of potential biomarkers for monitoring responses to MSC treatment and reveals a potential means of monitoring for other novel experimental treatments of sepsis.

Here, we show an increase in kynurenine and tryptophan in the serum of MSC-treated patients with septic shock. Tryptophan–kynurenine metabolism is activated in neurological disorders such as Alzheimer’s disease, Parkinson’s disease and schizophrenia, and it is associated with inflammatory diseases and cancer [17]. This pathway is associated with immunosuppression, a highly regulated check and balance immune system mechanism [17]. Kynurenine, synthesized by the enzyme tryptophan dioxygenase, is used in the production of niacin and suppresses the activity of proinflammatory cells and T-cell proliferation, modulates cytokine release [17] and can act as a neuroprotectant [25]. Kynurenine is also a source of NAD+ production, an important cofactor in energy metabolism [26]. Our analysis showed that indole-3 acetic acid (IAA) (Figure S6), a known antagonist of arylhydrocarbon receptors (AhR) involved in promoting vascular inflammation and oxidative stress, is significantly increased in MSC-treated patients [27]. An increase in indoleamine 2,3-dioxygenase (IDO), an enzyme that regulates tryptophan metabolism and its metabolites such as indole and indole acetic acid (increased in MSC-treated cohort), is associated with the inhibition of T-cell uptake due to MSC treatment [28]. MSC treatment on an animal model of sepsis showed the regulation of metabolites to levels similar to the sham control group. Aromatic amino acids (phenylalanine, tryptophan, tyrosine), L-methionine, thymine, threonine, betaine and homocysteine in MSC-treated animals with sepsis were adjusted to serum levels in sham controls. This study also showed decreases in TNF-a and IL-6 in the MSC-treated group that were similar to sham controls [29]. It has previously been shown that elevated kynurenine could potentially be a marker in sepsis and septic shock [30,31] and is correlated with poor outcomes and mortality [30,32]. In cases of severe sepsis and septic shock, treatment with GM-CSF has been associated with a reduction in plasma levels of kynurenine and tryptophan [33]. In the current study, increased kynurenine and its derivatives in patients treated with MSC may suggest the role of MSC treatment in upregulating kynurenine and may be a marker of a higher immune response among patients with septic shock.

However, our study lacks normal controls and nonseptic critically ill patients for comparison to further assess the metabolite alterations during septic shock and MSC treatment compared to uninfected or normal ICU controls. However, we show here that the metabolic phenotype of septic shock at baseline was similar to that of a previous study in our group of an independent cohort of patients with septic shock when it was compared to a noninfected ICU control cohort [34]. This may serve to validate the findings in the cohort with septic shock in this study.

Although MSC treatment is thought to reduce inflammation through innate and adaptive immune pathways, there are many controversies regarding immunomodulation and inflammatory stimulation by MSCs [35].

Of note, Schloesser et al. in 2019 [7] investigated the effect of MSC treatment on protein/cytokine profiles in the same patients used in our study, reporting no significant alteration in inflammatory biomarkers. These results provide supporting data for the hypothesis that MSCs may have a different mechanism of action on immunomodulatory effects, potentially through not-well-understood paracrine/endocrine signals, or perhaps a longer follow-up (>72 h) is needed to uncover inflammatory markers that were not seen in the first 72 h post-MSC therapy [36]. Nonetheless, in the present study, it appears that metabolomics is more reflective of changes seen in the serum of septic shock patients treated with MSCs within the first 72 h that are not seen in plasma inflammatory proteins/cytokines.

Here, both ^1^H-NMR and HILIC-MS analytical techniques were informative for monitoring metabolic response to MSC treatment within 72 h post-treatment. Although ^1^H-NMR is historically more consistent (specific), HILIC-MS is generally more sensitive than ^1^H-NMR; thus, MS-based metabolomics measurements may yield greater insight into the metabolic pathways affected by MSC treatment [37]. Importantly, we can use these preliminary metabolite results from this study showing MSC treatment effects (especially those with significant FDR changes) to help in a power analysis to calculate the sample size needed in a future phase II clinical trial of MSC treatment of septic shock patients to determine the number of patients needed to show a significant metabolomic response to MSC therapy. This sample size calculation would be based on the metabolomics effects seen rather than the outcome of treatment. However, the metabolic changes may reflect changes in outcomes in a larger, adequately powered phase II trial.

The major weakness of this study was the small sample size. With only nine MSC-treated septic shock patients and only three with low, three medium and three with high dose therapy cohorts, it is difficult to be definitive in our analysis. We used all the patient data available in the phase I study (21 septic shock controls and 9 MSC-treated septic shock patients) for our analysis; no other patient samples are available from this study. Although the sample number in this study is not ideal, we believe it yields important data and is the only study of metabolomics in MSC-treated septic shock patients that exists to our knowledge. External validation is strongly required to validate the preliminary metabolomics findings and help determine the potential of MSC therapy in the treatment of septic shock.

## 5. Conclusions

In this study, metabolomics investigation shows the impact of MSC therapy on metabolites in patients with septic shock within 72 h after treatment. While the phase I study showed no evidence of significant adverse effects of MSC therapy, nor significant changes in physiologic and clinical outcomes, nor systematic cytokine alterations, notably, plasma metabolomics measurement did reveal significant differences between the two cohorts. This means that metabolomics studies may be useful for monitoring treatment effects in future studies examining MSC treatment of septic shock patients and, thus, is highly recommended for any future phase II study.

## Figures and Tables

**Table 1:** OPLS-DA model characteristics to separate MSC-treated septic shock patients and nontreated septic shock controls at 24 h and 72 h (top panel) and at baseline (T = 0) (bottom panel). Importantly, the data at T = 0 strongly suggest no metabolomics difference between MSC-treated septic shock patients and nontreated septic shock controls at baseline. # shows the number of metabolites in the prediction models.

Separation of MSCs-Treated Cohort from Control Based on the Samples at 24 and 72 h								
Analytical Platform	R^2^Y	Q^2^Y	*p*-Value	Sensitivity	Specificity	AUROC	Metabolites #	PLS Regression (R^2^)
HILIC-MS	0.87	0.697	1.08 × 10^−7^	>99%	>99%	0.97	47	0.92
NMR	0.668	0.507	0.00015	91%	86%	0.85	17	0.89
**Separation of MSCs-Treated Cohort from Control Based on the Samples at Baseline (0H)**								
**Analytical Platform**	**R^2^Y**	**Q^2^Y**	***p*-Value**	**Sensitivity**	**Specificity**	**AUROC**	**Metabolites #**	**PLS Regression** **(R^2^)**
HILIC-MS	0.929	−0.16	1	-	-	-	47	-
NMR	0.519	−0.29	1	-	-	-	17	-

## Data Availability

Data are available on request from corresponding author. The data are not publicly available due to pending of the phase II study.

## Supplementary Materials

The following supporting information can be downloaded at: https://www.mdpi.com/article/10.3390/metabo13111142/s1, Additional File S1: Includes clarification of methods; Figure S1: PCA analysis shows clustering between MSC-treated septic shock patient samples compared to non-MSC- treated septic shock patient samples (age- and sex-matched at 24 and 72 h; no baseline samples are shown); Figure S2: PCA analysis of MSC-treated septic shock patients post-treatment compared to septic shock controls using the NMR dataset at (A) 24 h and (B) 72 h, showing clustering between cohorts using 56 metabolites (age- and sex-matched samples), and HILIC-MS dataset at (C) 24 h and, (D) 72 h, showing clustering between cohorts using 133 metabolites; Figure S3: PCA analysis of MSC-treated septic shock patients 24 and 72 h post-treatment compared to septic shock controls (this includes all controls; no age- and sex- matching) using the (A) HILIC-MS and (B) NMR datasets, showing a clustering between cohorts; Figure S4: PCA analyses on baseline samples using NMR (A) and HILIC-MS (B) datasets; Figure S5: PCA analyses on baseline samples (including all controls) using NMR (A) and HILIC-MS (B) datasets; Figure S6: The coefficient plot of 47 metabolites (with a VIP score of > 1.0) obtained by via HILIC-MS that have increased and decreased relative concentrations in the MSC-treated septic shock cohort vs. non-treated septic shock controls (age- and sex-matched samples at 24 h and 72 h); Figure S7: The coefficient plot of 17 metabolites (with a VIP score >1.0) obtained by via 1H-NMR that are increased and decreased in concentration in the MSC-treated septic shock cohort vs. the non-treated septic shock controls (age- and sex-matched samples at 24 h and 72 h); Figure S8: OPLS-DA analysis on the HILIC-MS dataset shows a robust separation between MSC-treated septic shock patient samples and non-MSC-treated septic shock controls using 51 metabolites (including all controls); Figure S9: OPLS-DA analysis of the 1H-NMR dataset at each time point (A) 24 h and (B) 72 h, showing the separation between the MSC-treated septic shock cohort compared to the untreated septic shock controls (age- and sex-matched samples at 24 h and 72 h); Figure S10: PCA analysis of A: HILIC-MS and B: NMR metabolomic datasets for high, medium, and low doses of MSC-treated septic shock patients; Figure S11: OPLS-DA on A: HILIC-MS and B:1H-NMR show higher metabolite alteration for the samples at 72 h than 24 h compared to baseline, as 24 h samples stay closer to the baseline data; Figure S12: OPLS-DA of the HILIC-MS dataset shows a significant difference between metabolites at 24 h and 72 h for the MSC-treated patients; Figure S14: Specific plasmaserum metabolite alterations over time in the HILIC-MS analysis show significant changes in metabolite concentrations at 72 h compared to 24 h and baseline levels in MSC-treated vs. non-MSC-treated septic shock patients; Figure S13: Permutation test for the OPLS-DA analysis on the separation of (A) the MSC-treated cohort from the non-MSC- treated septic shock cohort (HILIC-MS dataset), (B) the MSC-treated cohort from the non-MSC- treated septic shock cohort (NMR dataset), (C) lLow, medium and high doses of MSC treatment (HILIC-MS dataset), (D) samples 24 h and 72 h from baseline samples of MSC-treated cohort (HILIC-MS dataset); Figure S15: OPLS-DA analysis shows the separation between MSC-treated and non-MSC-treated using (A) HILIC-MS and (B) 1H-NMR, respectively; Table S1: Unpaired t-test analysis using HILIC-MS dataset between MSC-treated septic shock patient and non-MSC- treated septic shock patient plasmaserum samples combined at 24 and 72 h after infusion, showing 38 metabolites with *p* < 0.05 and 22 metabolites with (FDR (*q* value) < 0.05; Table S2: Unpaired t-test univariate analysis using the NMR dataset between MSCs-treated septic shock patients and non-treated septic shock cohort patient samples at 24 and 72 h combined post-infusion shows only 8 metabolites with significant differences (*p* < 0.05) and no metabolites with a significant FDR difference between cohorts; Table S3: Unpaired t-test of HILIC-MS data from MSC-treated septic shock patient serum samples at 72 h identified 48 metabolites significantly changed (*p*-value < 0.05); Table S4: ANOVA analysis of metabolites for high, medium, and low doses of MSC-treated patients, indicated 59 metabolites significantly changed among these groups, with 37 showing FDR differ-ences of < 0.05 between cohorts; Table S5: HILIC-MS data showing MSC-treated septic shock patients compared to non-MSC-treated septic shock patients at 24 h; Table S6: Pathway analysis shows upregulation and downregulation of biochemical pathways in MSC-treated septic shock patients when compared to non-MSC-treated septic shock control patients using the most differentiating metabolites of HILIC-MS and NMR data; References [7,8,9,10,11,12,13,14,15,16] are cited in the supplementary materials.
