# Supplementary material for: Metabolomics Analysis of Mesenchymal Stem Cell (MSC) Therapy in a Phase I Clinical Trial of Septic Shock: An Exploratory Study
Source: Metabolites. 2023 Nov 10;13(11):1142. doi: 10.3390/metabo13111142 (PMC10673547; doi:10.3390/metabo13111142)
Supplement: Supplementary file 1 [file metabolites-13-01142-s001.zip › metabolites-2671784-supplementary.pdf]

metabolites

MDPI

Supplementary (Figures and Tables)

Metabolomics Analysis of Mesenchymal Stem Cell (MSC) Therapy in a Phase I Clinical Trial of Septic Shock: An Exploratory Study

Mohammad M. Banoei [1], Lauralyn A. McIntyre [2,3,4], Duncan J. Stewart [3,5], Shirley H. J. Mei [3,6], David Courtman [3,6], Irene Watpool [3], John Granton [7], John Marshall [8], Claudia dos Santos [8], Keith R. Walley [9], Kenny Schlosser [3,6], Dean A. Fergusson [3,4] and Brent W. Winston [1,10], For the Canadian Critical Care Trials Group (CCCTG) [ŧ] and the Canadian Critical Care Translational Biology Group (CCCTBG) [ŧŧ]

Sample collection and enrollment

Serial serum samples were obtained from 30 patients at baseline (T = 0) prior to MSC infusion within 30 hours of admission to the ICU and 24 and 72 hours after MSC infusion for the treated group (n = 9) and control group (n = 21) without MSC infusion. Additional details have been provided on patient selection, treatment and the serum processing protocol, as previously described [7].

To minimize confounding factors, we took a common approach to control potential confounders [7] by age and sex matching; nine MSC-treated patients and nine age-matched (±2 y) and sex-matched nontreated controls were selected (from 21 non-MSC-treated patients) to establish multivariate and univariate statistical models of metabolomic analysis throughout the study.

Metabolite identification and profiling

For ^{1}H-NMR analysis, patient serum was filtered using 3kDa NanoSep microcentrifuge filters, and ^{1}H-NMR spectroscopy was performed using a 600 MHz NMR spectrometer (Bruker BioSpin Ltd., Milton, ON, Canada), as previously described [8]. NMR spectra were processed and profiled in a nontargeted approach using ChenomX NMR Suite 7.1 (ChenomX Inc., Edmonton, AB, Canada) [9,10]. Fifty-six metabolites were quantified using ^{1}H-NMR, including sugar alcohols, sugars, amino acids and volatile compounds.

For HILIC-MS, metabolites were extracted from serum using 50% methanol solvent followed by centrifugation and transferring supernatant to the mass spectrometer. A UHPLC-MS (Q Exactive HF Hybrid Quadrupole-Orbitrap Mass Spectrometer, Thermo-Fisher, Calgary, AB, Canada) was used to analyze the serum samples. We used an ultra-high-performance liquid chromatography system using a 2.1mm x 100mm long Syncronis HILIC (Thermo-Fisher, Calgary, AB, Canada) LC column that was packed in-house with 3µm pore Hypercarb particles. More details about HILIC-MS analysis can be found below. Maven software (El Maven v12.0) was used to process HILIC-MS spectral data for the identification and quantification of metabolites [11,12]. Using an untargeted approach, 133 metabolites were quantified using HILIC-MS and consisted of amino acids, organic acids, sugars, sugar alcohols and acylcarnitines compounds.

Data Analysis

Both univariate and multivariate data analyses were applied to extract information from the metabolomic datasets. Univariate analyses were used as complementary methods for multivariate analysis to provide more information on metabolomic profiles as well as each metabolite individually. MetaboAnalyst 5.0 [14] and MetaBox v1.2 [13] were used for univariate data analyses. Data were not preprocessed (normalization, scaling and transformation) for the univariate analyses. The majority of metabolomics datasets were

not normally distributed (prior to processing) based on the Shapiro–Wilk test. Principal component analysis (PCA) was performed using datasets derived from serum samples to evaluate metabolite inter-relationships and the aggregation of cohorts using the metabolomics dataset between MSCs-treated and non-MSC-treated control cohorts.

Partial least squares discriminant analysis (PLS-DA) and orthogonal partial least squares discriminant analysis (OPLS-DA) were used to separate populations by metabolic profiles based on the most differentiating metabolites. Q^2Y and cross-validation ANOVA (CV-ANOVA) parameters were considered for predictability and significance of the separation of populations based on the metabolomics profiles (see section below). A variable importance in the projection (VIP) approach was applied to streamline the metabolite list and reduce the complexity of a large number of differentiating metabolites. We also applied S-plot and coefficient plot analyses to look at the metabolites that contributed most to the separation of the variables in the study. S-plot was used to extract the putative biomarkers that have both high reliability and magnitude. Compounds with covariance > 0.1 were considered important metabolites. SIMCA-P v 15.0.2 (Umetrics AB, Umeå, Sweden) and MetaboAnalyst 5.0 software (Mcgill University, Montreal, Canada) were used for multivariate analyses. Data were normalized (median), transformed (log) and autoscaled (i.e., processed) for multivariate analyses. Partial least squares regression (PLSR) was applied to show the relation of the most important metabolites obtained via OPLS-DA and PLS-DA analysis to the separation of the cohorts. Permutation tests were performed to check the validity of prediction (Q^2) and to prevent potential overfit for all OPLS-DA and PLS-DA analyses using 200 random permutation tests. A misclassification table was obtained for the predicting group (randomly selected ¼ of samples, three-times repetition) using SIMCA-P v15.0.2, (Mcgill University, Montreal, Canada) and AUROC was calculated using Graph Pad Prism v3.03 (Boston, MA, USA). Parallel pathway analyses were performed using MetaboAnalyst 4.0 [15], a free web-based tool, and Cytoscape 3.6.0 (NIGMS, Bethesda, MD, USA) [16], using the most differentiating metabolites involved in the separation of the MSC-treated cohort compared to the non-MSC-treated controls.

The website for members of the Canadian Critical Care Trials Group and the Canadian Critical Care Translational Biology Group can be found at www.ccctg.ca.

References

7. Schlosser, K.; Wang, J.-P.; dos Santos, C.; Walley, K.R.; Marshall, J.; Fergusson, D.A.; Winston, B.W.; Granton, J.; Watpool, I.; Stewart, D.J.; et al. Effects of Mesenchymal Stem Cell Treatment on Systemic Cytokine Levels in a Phase 1 Dose Escalation Safety Trial of Septic Shock Patients. *Crit. Care Med.* **2019**, *47*, 918–925
8. Banoei, M.M.; the Canadian Critical Care Translational Biology Group (CCCTBG); Vogel, H.J.; Weljie, A.M.; Kumar, A.; Yende, S.; Angus, D.C.; Winston, B.W. Plasma metabolomics for the diagnosis and prognosis of H1N1 influenza pneumonia. *Crit. Care* **2017**, *21*, 97.
9. Weljie, A.M.; Dowlatabadi, R.; Miller, B.J.; Vogel, H.J.; Jirik, F.R. An Inflammatory Arthritis-Associated Metabolite Biomarker Pattern Revealed by1H NMR Spectroscopy. *J. Proteome Res.* **2007**, *6*, 3456–3464.
10. Weljie, A.M.; Newton, J.; Mercier, P.; Carlson, E.; Slupsky, C.M. Targeted profiling: quantitative analysis of 1H NMR metabolomics data. *Anal Chem.* **2006**, *78*, 4430–4442.
11. Clasquin, M.F.; Melamud, E.; Rabinowitz, J.D. LC-MS data processing with MAVEN: A metabolomic analysis and visualization engine. *Curr. Protoc. Bioinform.* **2012**, *37*, 14–11.
12. Melamud, E.; Vastag, L.; Rabinowitz, J.D. Metabolomic Analysis and Visualization Engine for LC−MS Data. *Anal. Chem.* **2010**, *82*, 9818–9826.
13. Xia, J.; Sinelnikov, I.V.; Han, B.; Wishart, D.S. MetaboAnalyst 3.0—making metabolomics more meaningful. Nucleic acids research 2015, *43*, W251–W257.
14. Wanichthanarak, K.; Fan, S.; Grapov, D.; Barupal, D.K.; Fiehn, O. Metabox: A Toolbox for Metabolomic Data Analysis, Interpretation and Integrative Exploration. *PLOS ONE* **2017**, *12*, e0171046
15. Xia, J.; Wishart, D.S. Using MetaboAnalyst 3.0 for Comprehensive Metabolomics Data Analysis. *Curr. Protoc. Bioinform.* **2016**, *55*, 14.10.1–14.10.91.
16. Shannon, P.; Markiel, A.; Ozier, O.; Baliga, N.S.; Wang, J.T.; Ramage, D.; Amin, N.; Schwikowski, B.; Ideker, T. Cytoscape: A software environment for integrated models of Biomolecular Interaction Networks. *Genome Res.* **2003**, *13*, 2498–2504.

Figures and Tables

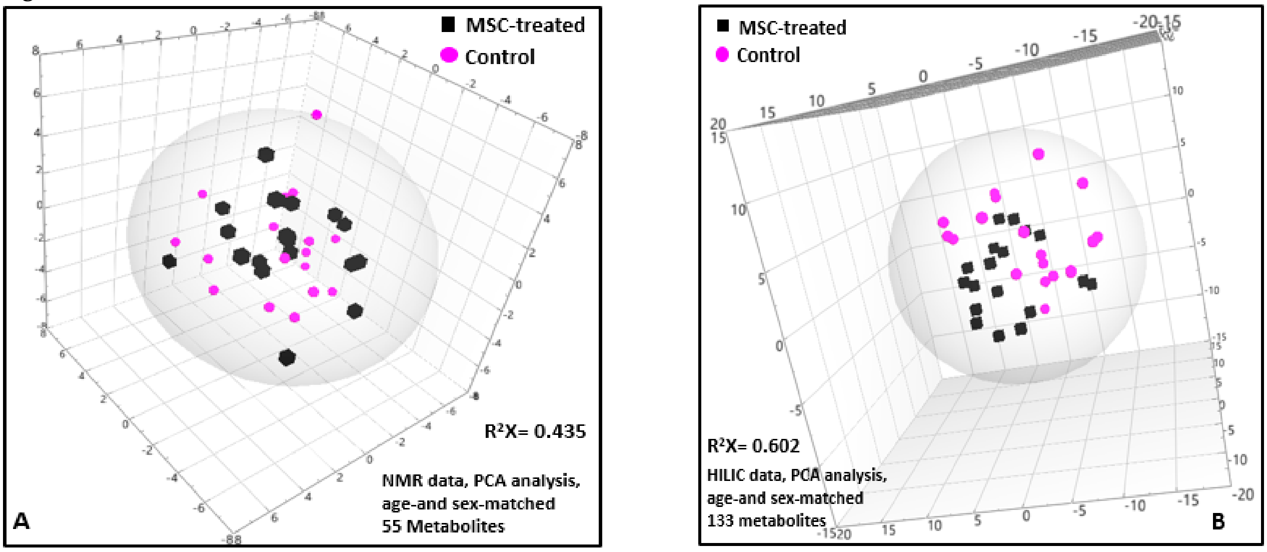

Figure S1. PCA analysis shows clustering between MSC-treated septic shock patient samples compared to non-MSC-treated septic shock patient samples (age- and sex-matched at 24 and 72 h; no baseline samples are shown). (**A**) NMR, (**B**) HILIC-MS.

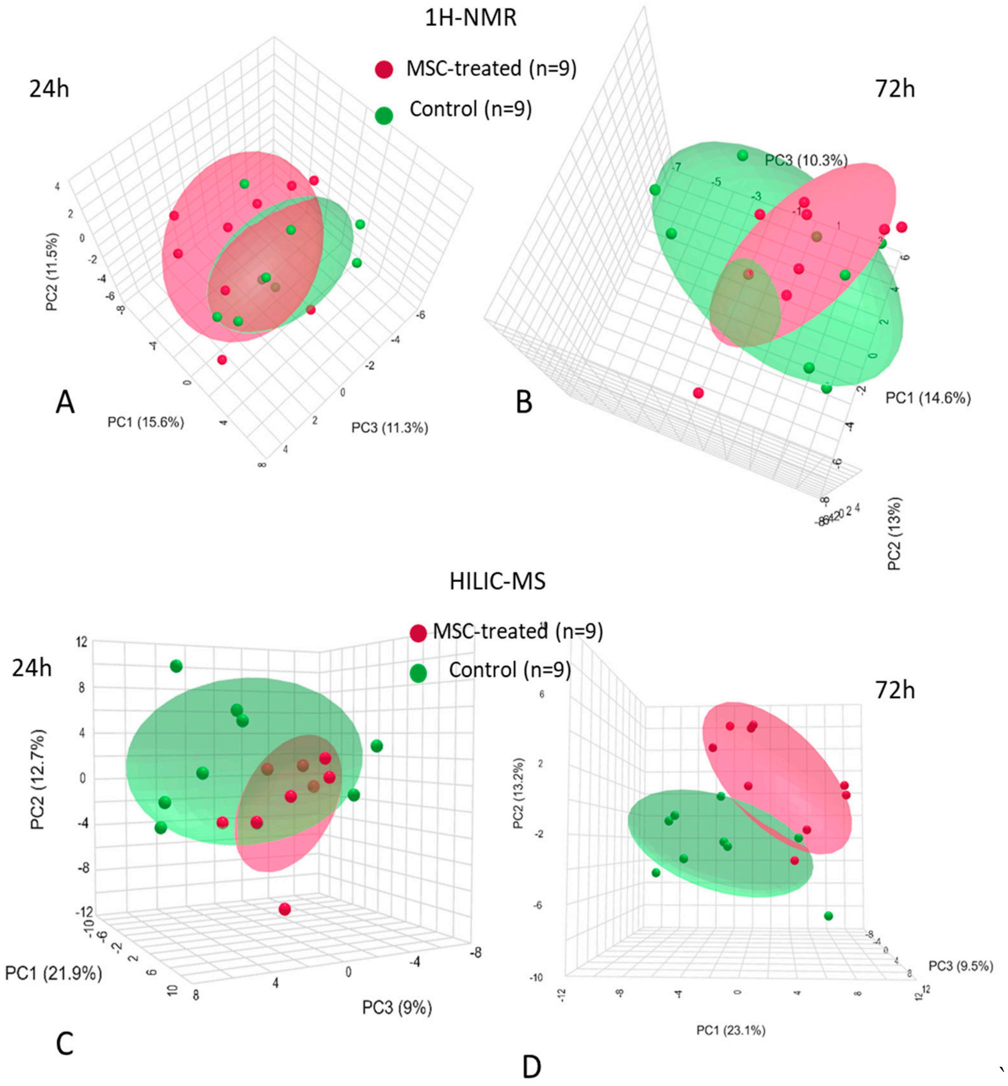

Figure S2. PCA analysis of MSC-treated septic shock patients post-treatment compared to septic shock controls using the NMR dataset at (**A**) 24 h and (**B**) 72 h, showing clustering between cohorts using 56 metabolites (age- and sex-matched samples), and HILIC-MS dataset at (**C**) 24 h and (**D**) 72 h, showing clustering between cohorts using 133 metabolites.

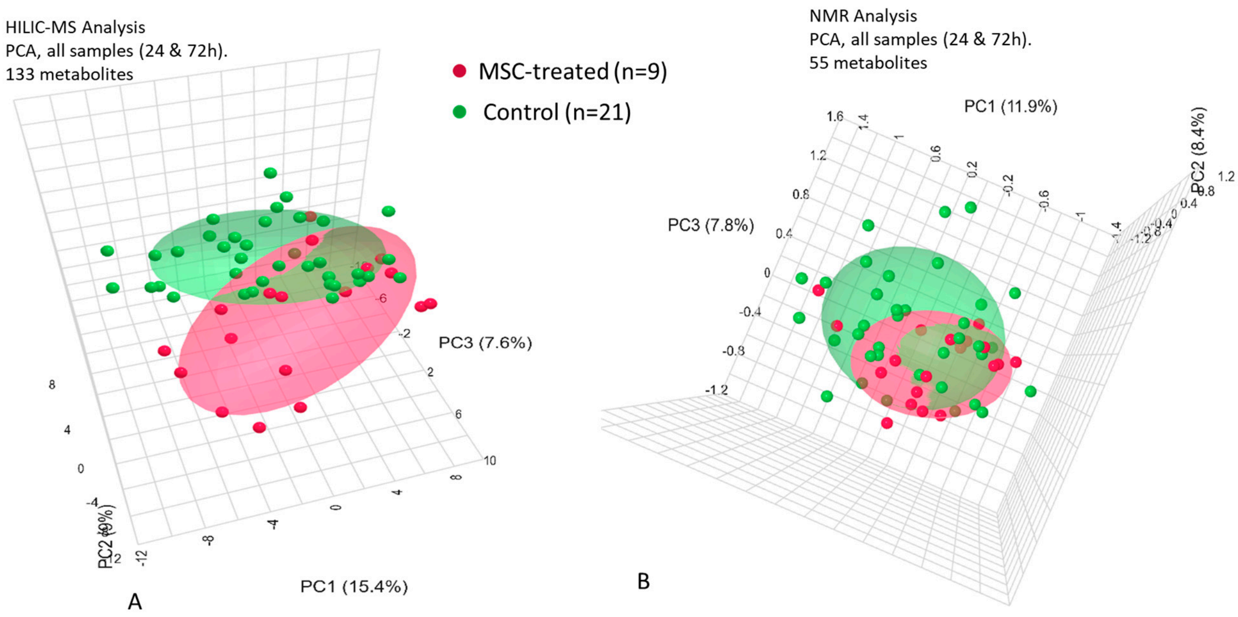

Figure S3. PCA analysis of MSC-treated septic shock patients 24 and 72 h post-treatment compared to septic shock controls (this includes all controls; no age- and sex-matching) using the (A) HILIC-MS and (B) NMR datasets, showing a clustering between cohorts.

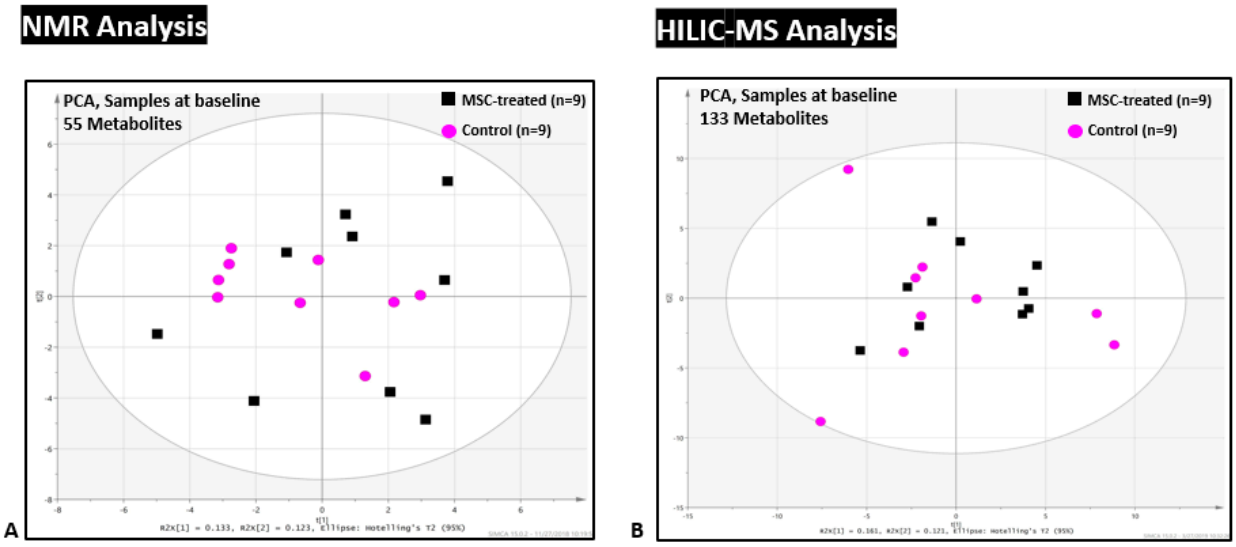

Figure S4. PCA analyses on baseline samples using NMR (**A**) and HILIC-MS (**B**) datasets. There is no clustering or grouping among samples at baseline for the two cohorts (age- and sex-matched samples), strongly suggesting there are no differences between the cohorts at baseline.

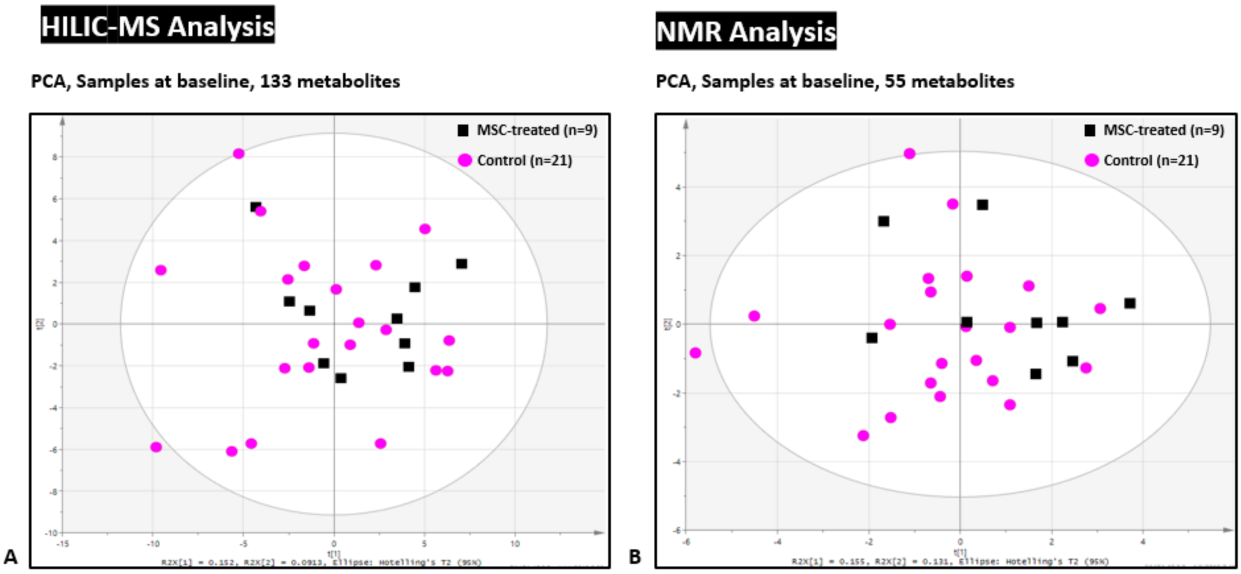

Figure S5. PCA analyses on baseline samples (including all controls) using NMR (**A**) and HILIC-MS (**B**) datasets. There is no clustering or grouping among samples at baseline for the two cohorts, strongly suggesting there are no differences between the cohorts at baseline even when all controls are used for comparison.

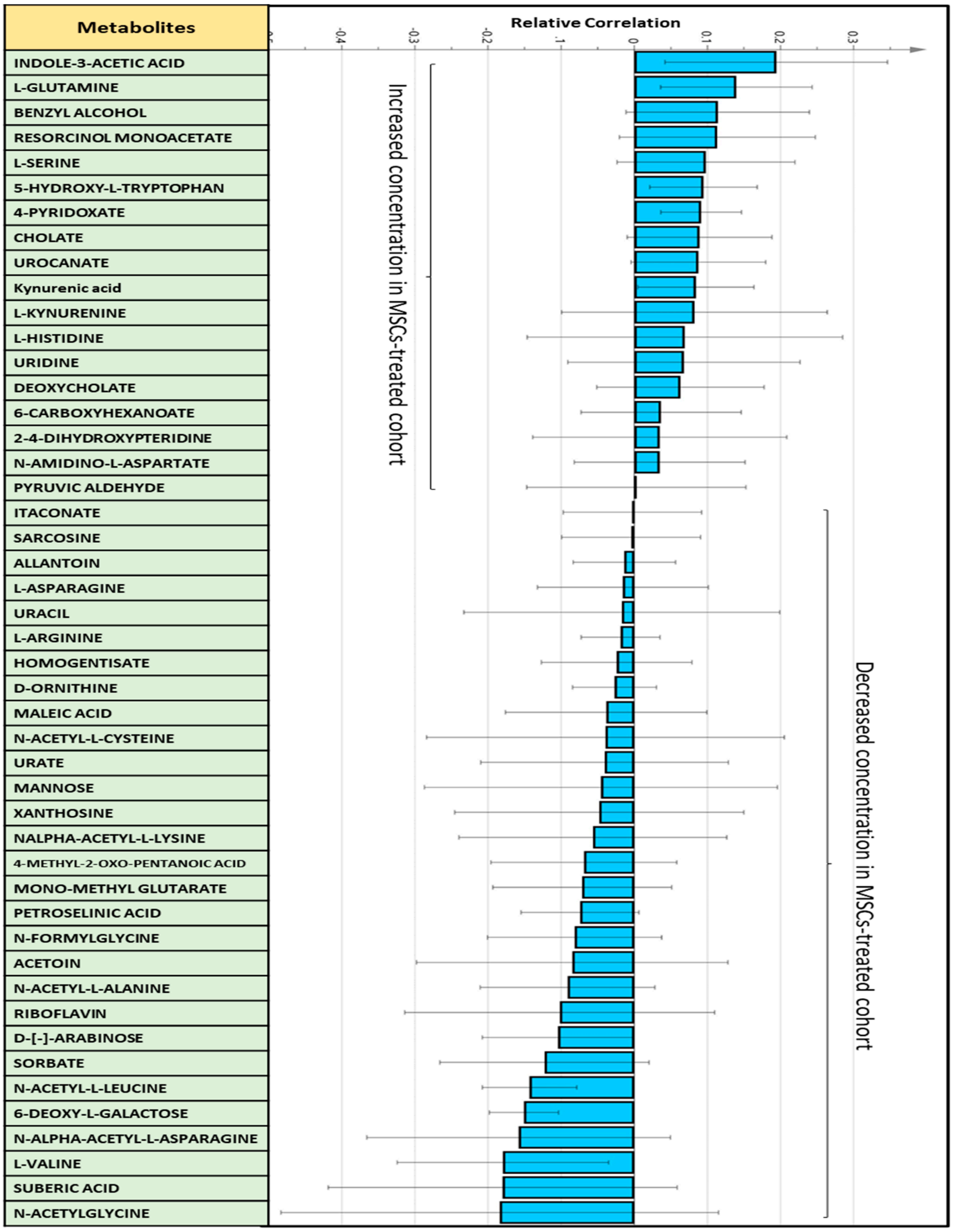

Figure S6. The coefficient plot of 47 metabolites (with a VIP score of > 1.0) obtained via HILIC-MS that have increased and decreased relative concentrations in the MSC-treated septic shock cohort vs. nontreated septic shock controls (age- and sex-matched samples at 24 h and 72 h).

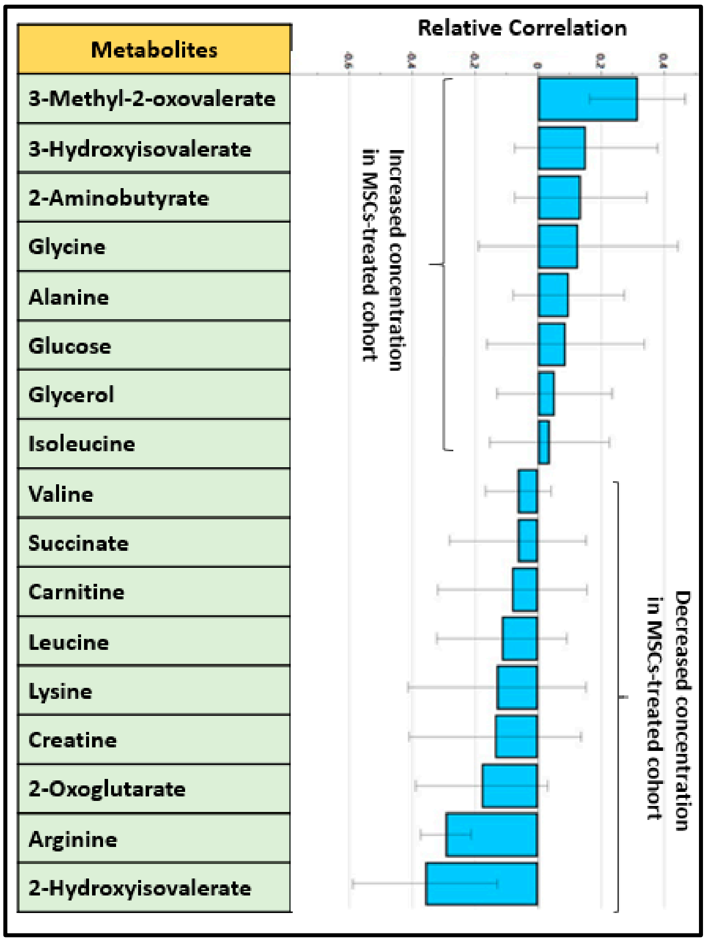

Figure S7. The coefficient plot of 17 metabolites (with a VIP score >1.0) obtained via ^{1}H-NMR that are increased and decreased in concentration in the MSC-treated septic shock cohort vs. the non-treated septic shock controls (age- and sex-matched samples at 24 h and 72 h).

HILIC-MS Analysis

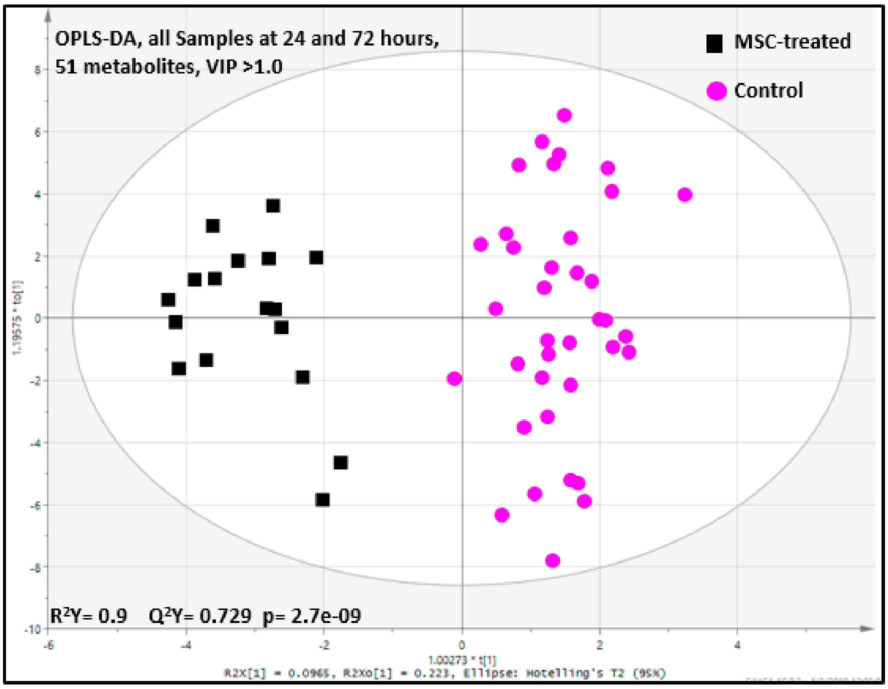

Figure S8. OPLS-DA analysis on the HILIC-MS dataset shows a robust separation between MSC-treated septic shock patient samples and non-MSC-treated septic shock controls using 51 metabolites (including all controls). The R2X is 0.9, suggesting a good fit for the data. The $Q^2Y = 0.729$ and *p*-value ($p = 2.7 \times 10^{-9}$) indicate a significant predictive model.

NMR Analysis

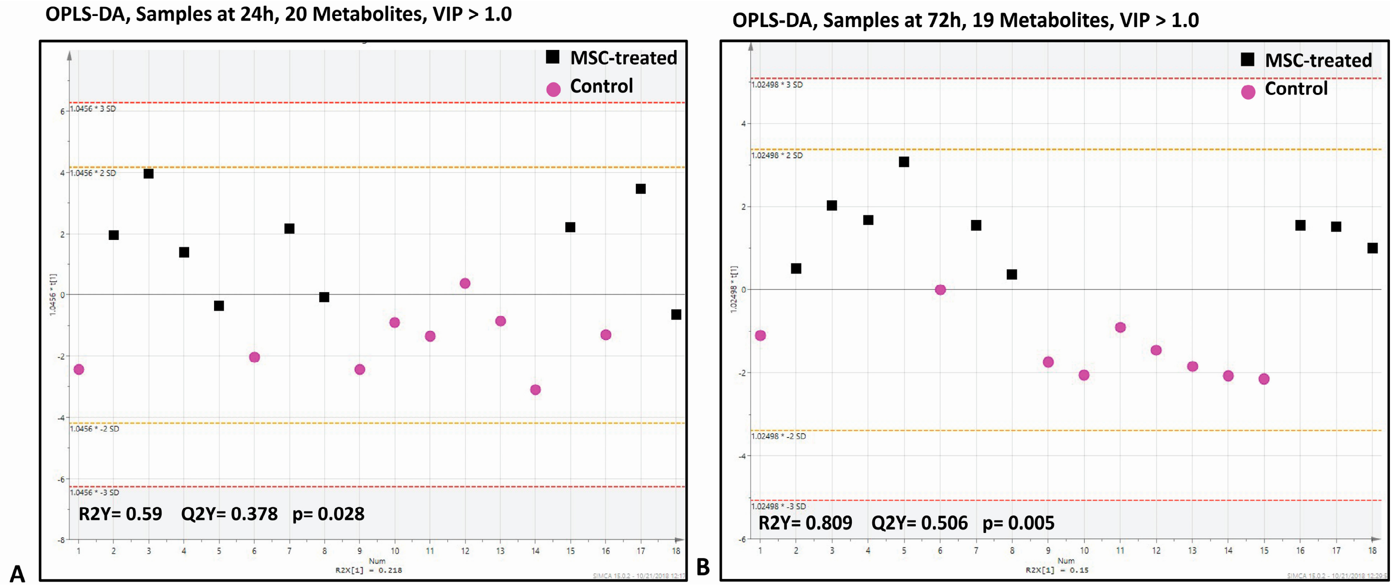

Figure S9. OPLS-DA analysis of the ^{1}H-NMR dataset at each time point (**A**) 24 h and (**B**) 72 h, showing the separation between the MSC-treated septic shock cohort compared to the untreated septic shock controls (age- and sex-matched samples at 24 h and 72 h).

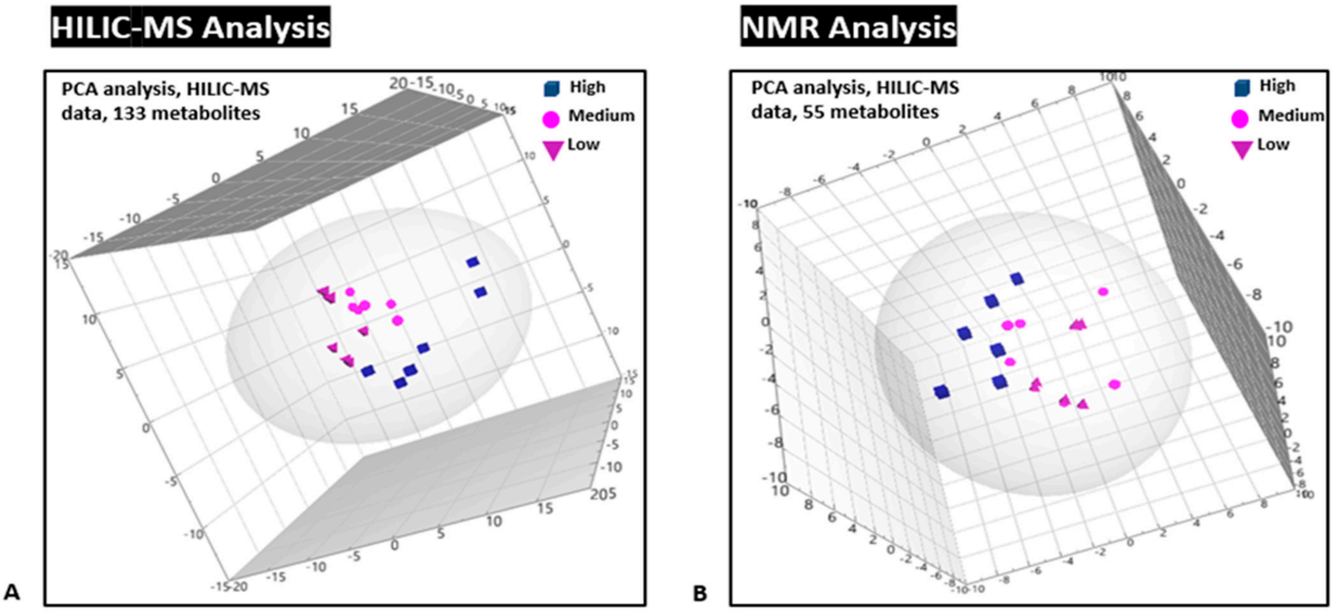

Figure S10. PCA analysis of **A**: HILIC-MS and **B**: NMR metabolomic datasets for high, medium and low doses of MSC-treated septic shock patients. There is grouping among the patient cohorts due to dose-dependent perturbations in metabolic profiles.

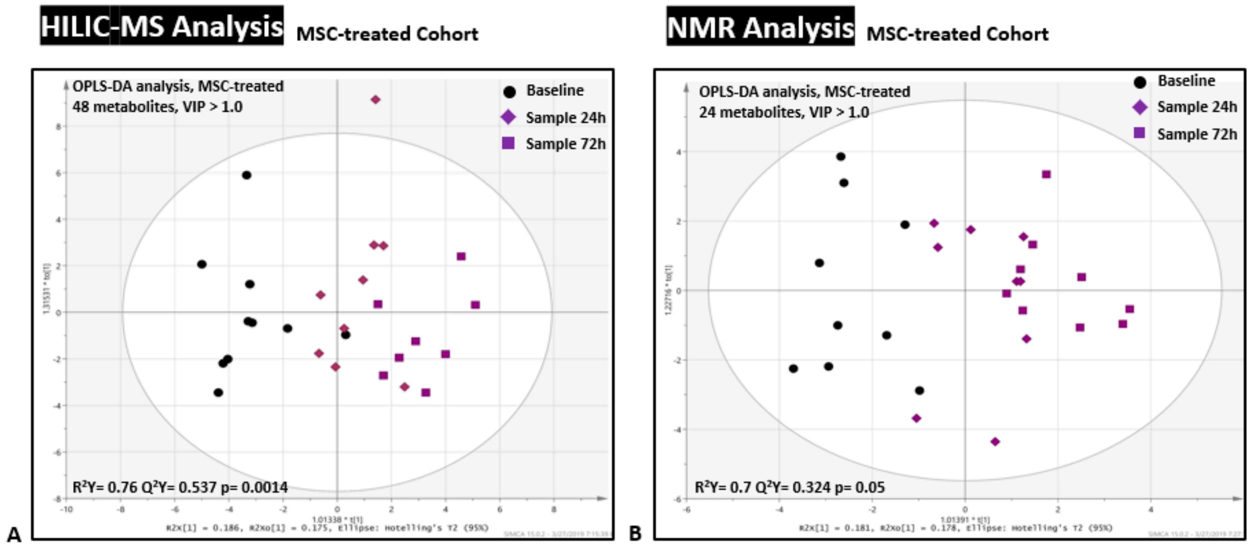

Figure S11. OPLS-DA on **A**: HILIC-MS and **B**:^{1}H-NMR show higher metabolite alteration for the samples at 72 h than 24 h compared to baseline, as 24 h samples stay closer to the baseline data.

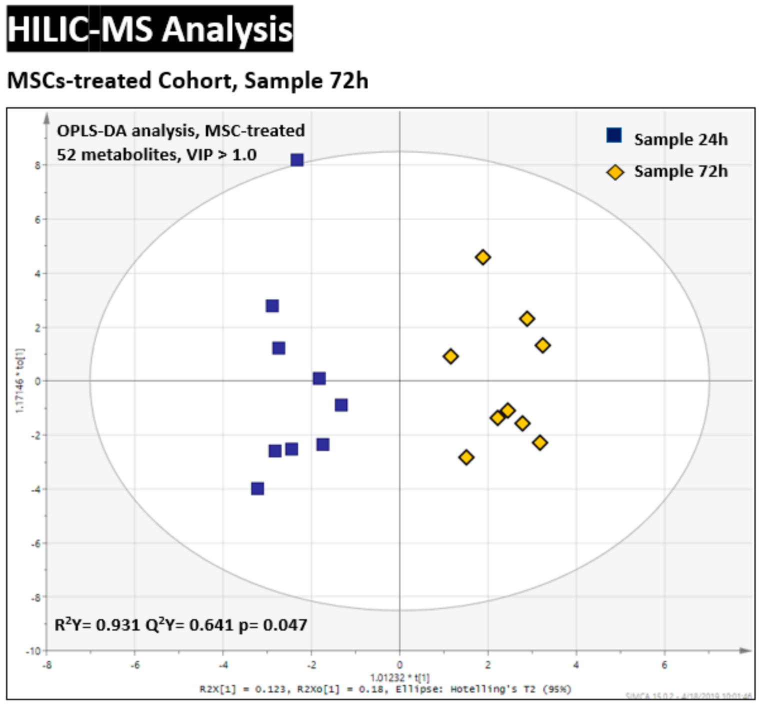

Figure S12. OPLS-DA of the HILIC-MS dataset shows a significant difference between metabolites at 24 h and 72 h for the MSC-treated patients.

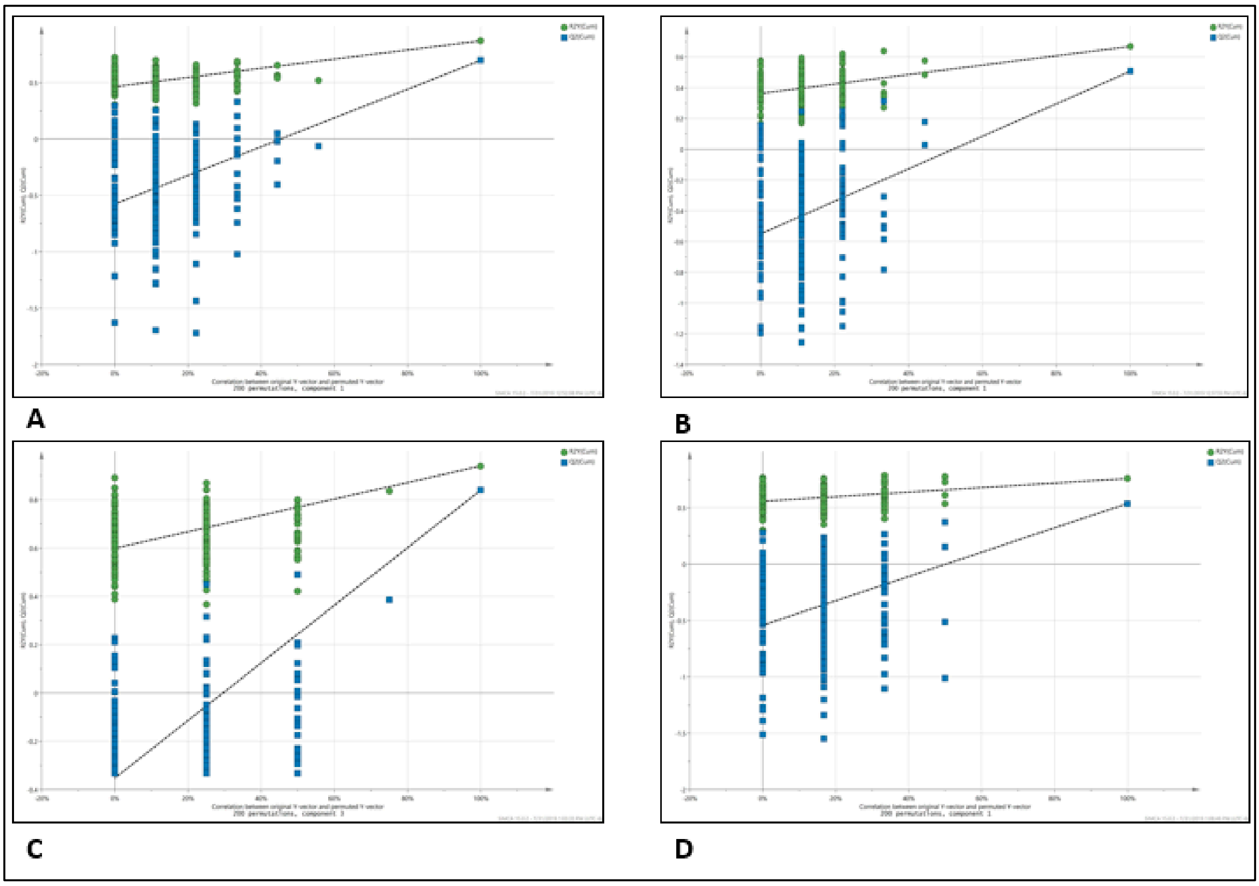

Figure S13. Permutation test for the OPLS-DA analysis on the separation of (**A**) the MSC-treated cohort from the non-MSC-treated septic shock cohort (HILIC-MS dataset); (**B**) the MSC-treated cohort from the non-MSC-treated septic shock cohort (NMR dataset); (**C**) low, medium and high

doses of MSC treatment (HILIC-MS dataset); (**D**) samples 24 h and 72 h from baseline samples of MSC-treated cohort (HILIC-MS dataset).

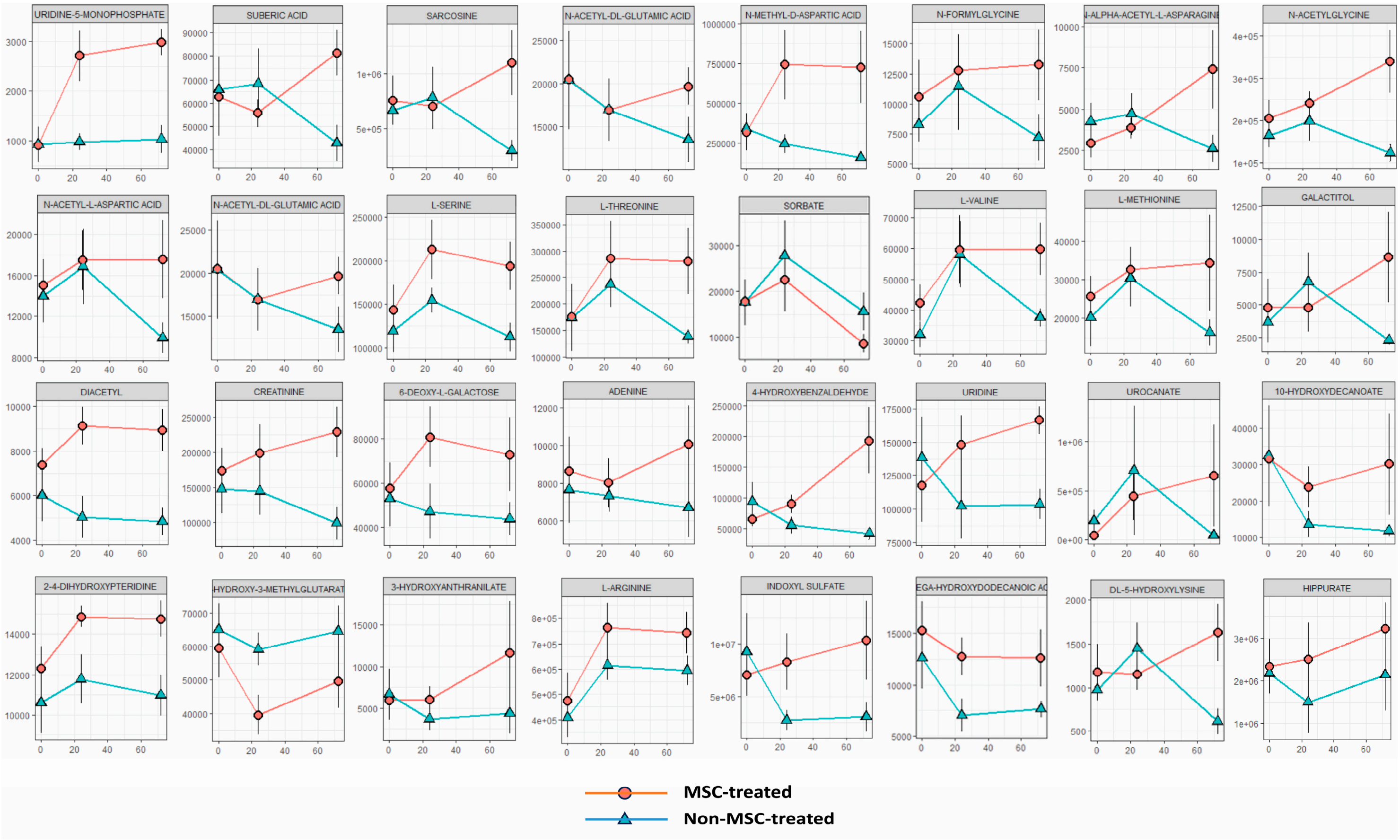

Figure S14. Specific serum metabolite alterations over time in HILIC-MS analysis show significant changes in metabolite concentrations at 72 h compared to 24 h and baseline levels in MSC-treated vs. non-MSC-treated septic shock patients.

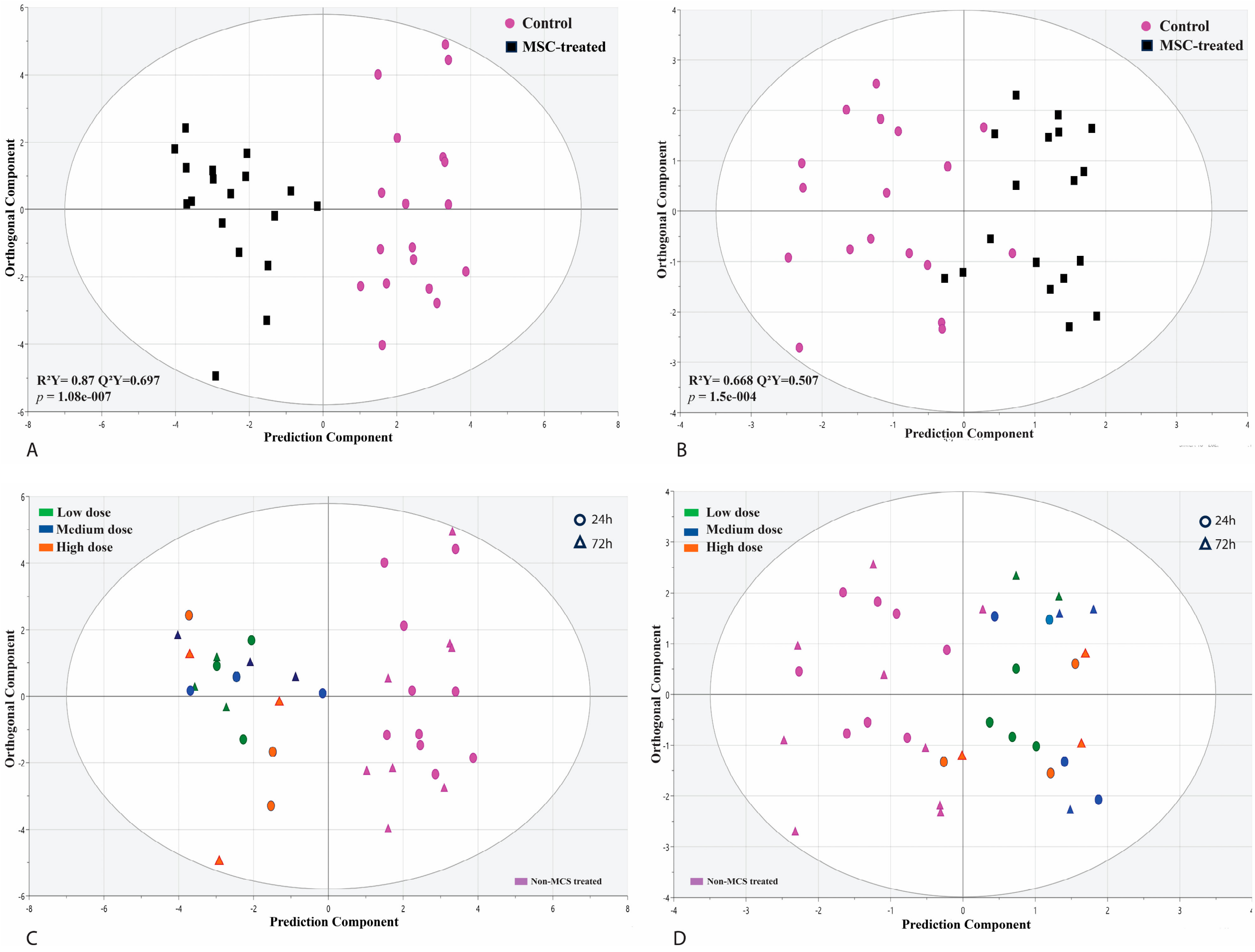

Figure S15. OPLS-DA analysis shows the separation between MSC-treated and non-MSC-treated using (**A**) HILIC-MS and (**B**) 1H-NMR, respectively. The same OPLS-DA plots represent the samples at 24 and 72 h for both groups and indicate the different doses of MSC-treated group using (**C**) HILIC-MS and (**D**) 1H-NMR.

Table S1. Unpaired t-test analysis using HILIC-MS dataset between MSC-treated septic shock patient and non-MSC-treated septic shock patient serum samples combined at 24 and 72 h after infusion, showing 38 metabolites with p < 0.05 and 22 metabolites with (FDR (q value) < 0.05. The values of metabolites are in ion intensity and relative concentration. W: Wilkson–Mann–Whitney analysis.

	Name	Mean (SD) of MSC-treated cohort 24 and 72 h	Mean (SD) of non-MSC-treated cohort 24 and 72 h	*p*-value	*q*-value (FDR)	Fold change	MSC-treated /non-MSC-treated
1	**L-Serine**	203,338 (90,707)	133,275 (49,911)	0.008	0.0445	1.53	Up
2	**6-Deoxy-L-Galactose**	76,955 (44,919)	45,351 (29,739)	0.0179	0.0708	1.7	Up
3	**Xanthosine**	6983 (4442)	3,948 (2,559)	0.0183	0.0708	1.77	Up
4	**L-Arginine**	754,189 (257,407)	604,131 (172,219)	0.0476	0.148	1.25	Up
5	**Uridine-5-Monophosphate**	2,843 (1,191)	1,001 (677)	<0.0001	0.0001	2.84	Up
6	**Diacetyl**	19,040 (26,260)	49,395 (23,200)	<0.0001	0.0014	1.83	Up
7	**4-Hydroxybenzaldehyde**	141860 (128,032)	48,914 (34,186)	<0.0001 (W)	0.0014	2.9	Up
8	**Azelaic Acid**	115,348 (60,437)	67,723 (72,531)	0.0002 (W)	0.0052	1.7	Up
9	**3-Hydroxy-3-Methylglutarate**	44,585 (21,165)	61,943 (18844)	0.0002 (W)	0.0059	-1.39	Down
10	**Uridine**	157,308 (50,881)	102,898 (55,740)	0.0004 (W)	0.01	1.53	Up
11	**Mono-Methyl Glutarate**	164,865 (175,203)	68,120 (70,533)	0.0005 (W)	0.01	2.42	Up
12	**N-Acetylglycine**	290,917 (172,343)	161,428 (110,379)	0.0008 (W)	0.0118	1.8	Up
13	**2-4-Dihydroxypteridine**	14,806 (2,129)	11,385 (3,269)	0.0008 (W)	0.0118	1.3	Up
14	**N-Methyl-D-Aspartic Acid**	734,783 (650,575)	200,958 (142,853)	0.0016 (W)	0.0209	3.66	Up
15	**Homogentisate**	11,835 (19,261)	3,035 (6,495)	0.0016 (W)	0.0209	3.9	Up
16	**Resorcinol Monoacetate**	831,359 (606,257)	319,725 (468,228)	0.00273 (W)	0.0253	2.6	Up
17	**Benzyl Alcohol**	40,608 (30,215)	17,176 (24,531)	0.0026 (W)	0.0264	2.36	Up
18	**L-Tyrosine**	66,368 (45,226)	44,582 (35,283)	0.0037 (W)	0.0347	1.49	Up
19	**Omega-Hydroxydodecanoic Acid**	12,677 (6,762)	7,351 (3,701)	0.0052 (W)	0.039	1.72	Up
20	**Uracil**	12,053 (5,381)	8,402 (6481)	0.0058 (W)	0.039	1.43	Up
21	**Alpha-Hydroxybutyric Acid**	9,968,711 (11,255,757)	3,582,864 (3,585,487)	0.0058 (W)	0.039	2.78	Up
22	**6-Carboxyhexanoate**	112,824 (78,079)	62,555(81347)	0.0064 (W)	0.039	1.8	Up
23	**Urocanate**	547,448 (1,181,664)	372,869 (1,404,004)	0.0064 (W)	0.039	1.47	Up
24	**N-Acetyl-L-Alanine**	370,371 (161,975)	245,939. (157,859)	0.0064 (W)	0.039	1.51	Up
25	**Urate**	24,494,444 (9,535,412)	16,742,740 (9,290,220)	0.0086 (W)	0.0465	1.46	Up
26	**Allantoin**	679,500 (574,500)	421,867 (513,513)	0.0106 (W)	0.0551	1.61	Up
27	**Acetoin**	15,536 (5,125)	11,382 (7,740)	0.0117 (W)	0.0586	1.36	Up
28	**L-Tryptophan**	11,655 (9,521)	7,195 (6,917)	0.0129 (W)	0.0602	1.62	Up

29	**3-Hydroxyanthranilate**	8,855 (13,310)	4031 (5,861)	0.0129 (W)	0.0602	2.2	Up
30	**10-Hydroxydecanoate**	26,959 (30,955)	12,798 (8,311)	0.0156 (W)	0.0703	2.11	Up
31	**Glycolaldehyde Dimer**	327,422 (472,934)	90,539 (135,854)	0.0187 (W)	0.0708	3.62	Up
32	**Creatinine**	213,258 (114,288)	121,982 (88,296)	0.0187 (W)	0.0708	1.75	Up
33	**Indoxyl Sulfate**	9,297,576 (9,592,629)	2,877,781 (3,509,477)	0.0187 (W)	0.0708	3.23	Up
34	**N-Formylglycine**	13,032 (8,611)	9,315 (8,812)	0.0224 (W)	0.0803	1.4	Up
35	**D-Pantothenic Acid**	26,462 (21,992)	15,759 (22,317)	0.0224 (W)	0.0803	1.68	Up
36	**Methyl Vanillate**	17,267 (6,966)	12,131 (7,764)	0.0266 (W)	0.0931	1.42	Up
37	**Glycocholate**	130,418 (119,336)	59,388 (104,649)	0.0402 (W)	0.1309	2.2	Up
38	**4-Acetamidobutanoate**	411,117 (425,864)	182,515 (206,763)	0.0444 (W)	0.1412	2.25	Up

Table S2. Unpaired t-test univariate analysis using the NMR dataset between MSCs-treated septic shock patients and nontreated septic shock cohort patient samples at 24 and 72 h combined postinfusion shows only 8 metabolites with significant differences (p< 0.05) and no metabolites with a significant FDR difference between cohorts. The values of metabolites are in µM concentration.

	**Name**	**Mean (SD) of MSC treated cohort 24h and 72h**	**Mean (SD) of MSC treated cohort 24h and 72h**	***p*-value**	***q*-value (FDR)**	**Fold change**	**MSC-treated/Non MSC-treated**
1	2-Oxoglutarate	0.037 (0.017)	0.051 (0.018)	0.0234	0.3886	-1.38	Down
2	Proline	0.072 (0.026)	0.090 (0.031)	0.0601	0.421	-1.26	Down
3	2-Hydroxyisovalerate	0.007 (0.006)	0.014 (0.011)	0.0129 (W)	0.3886	-1.96	Down
4	Leucine	0.043 (0.019)	0.057 (0.020)	0.0235 (W)	0.3886	-1.3	Down
5	Succinate	0.006 (0.006)	0.011 (0.016)	0.0287 (W)	0.3886	-1.86	Down
6	Creatine	0.118 (0.056)	0.213 (0.139)	0.0379 (W)	0.3886	-1.81	Down
7	Histidine	0.026 (0.005)	0.032 (0.010)	0.0442	0.3886	-1.24	Down
8	Lysine	0.056 (0.021)	0.073 (0.027)	0.0486	0.3886	-1.29	Down

Table S3. Unpaired t-test of HILIC-MS data from MSC-treated septic shock patient serum samples at 72 h identified 48 metabolites significantly changed (p-value < 0.05). A total of 21 metabolites show FDR < 0.05 significant differences between MSC-treated septic shock patients when compared to non-MSC-treated septic shock controls.

	**Name**	**Mean (SD) of MSC-treated cohort 72 h**	**Mean (SD) of non-MSC-treated 72 h**	***p*-value**	***q*-value (FDR)**	**Fold change**	**MSC-treated/non-MSC-treated**
**1**	**Uridine-5-Monophosphate**	2,979 (786)	1,030 (840)	0.0001	0.007	2.89	Up
**2**	**Uridine**	166,619 (29,795)	103,583 (33,094)	0.0006	0.0198	1.61	Up
**3**	**L-Tyrosine**	55,716 (17,931)	28,895 (12988)	0.0022	0.027	1.93	Up
**4**	**Diacetyl**	8,943 (2,844)	4,839 (1,878)	0.0023	0.027	1.85	Up
**5**	**N-Acetyl-L-Alanine**	374,065 (135975)	204,360 (71,626)	0.0044	0.0398	1.83	Up
**6**	**5-Hydroxy-L-Tryptophan**	6,924 (1,972)	3,559 (2,421)	0.0052	0.0398	1.95	Up
**7**	**Suberic Acid**	81,468 (29,105)	42,906 (23,695)	0.0071	0.0449	1.9	Up
**8**	**Creatinine**	228,384 (108,764)	99,273 (70,342)	0.0087	0.0478	2.3	Up
**9**	**L-Tryptophan**	10,939 (4,892)	5,218 (3,618)	0.0123	0.0652	2.1	Up

**10**	**2-4-Dihydroxypteridine**	14,748 (2,674)	10,985 (3,044)	0.0132	0.0657	1.34	Up
**11**	**Xanthosine**	8,157 (4,622)	3,181 (2,182)	0.0135	0.0657	2.56	Up
**13**	**Glycocholate**	124,932 (103,888)	21,134 (21,530)	0.0173	0.0769	5.91	Up
**14**	**L-Serine**	193,656 (83,167)	112,164 (48,954)	0.0221	0.0862	1.73	Up
**15**	**Urate**	24,211,111 (10,522,172)	14,508,966 (4,998,346)	0.0237	0.0862	1.67	Up
**16**	**Hypotaurine**	2,254 (1,242)	3,883 (1,521)	0.0243	0.0862	-1.72	Down
**17**	**N-Acetyl-D-Mannosamine**	12,539 (5,188)	7,396 (3,693)	0.0277	0.0885	1.7	Up
**18**	**D-Ribose**	4,321 (2,919)	1,744 (1,391)	0.0295	0.0885	2.48	Up
**19**	**D--Galacturonic Acid**	13990 (10424)	4,893 (2,237)	0.0314	0.0885	2.86	Up
**20**	**L-Valine**	59,946 (25,585)	37,453 (8,781)	0.0321	0.0885	1.6	Up
**21**	**Methyl Vanillate**	16,886 (7,032)	10,704 (4,900)	0.0459	0.1215	1.58	Up
**22**	**Azelaic Acid**	114,847 (71,325)	42356 (10,867)	<0.0001 (W)	0.007	2.71	Up
**23**	**Homogentisate**	17,160 (25,720)	1,107 (1,424)	0.0008 (W)	0.0198	15.5	Up
**24**	**4-Hydroxybenzaldehyde**	193,549 (163,858)	42,293 (28,773)	0.0008 (W)	0.0198	4.58	Up
**25**	**N-Acetylglycine**	340,460 (224,639)	123,851 (61,611)	0.0012 (W)	0.026	2.75	Up
**26**	**N-Methyl-D-Aspartic Acid**	727,571 (682,976)	156,411 (69,385)	0.0019 (W)	0.0261	4.65	Up
**27**	**6-Carboxyhexanoate**	119,661 (83,861)	41,245 (65,385)	0.0019 (W)	0.0261	2.9	Up
**28**	**Allantoin**	724,428 (555,105)	251,265 (79,324)	0.0019 (W)	0.0261	2.88	Up
**31**	**Mono-Methyl Glutarate**	122,394 (53,428)	51,917 (39,200)	0.0056 (W)	0.0398	2.36	Up
**32**	**Urocanate**	652,371 (1,563,387)	41,160 (66,790)	0.0056 (W)	0.0398	15.85	Up
**33**	**Resorcinol Monoacetate**	774,846 (378,753)	213,466 (349,473)	0.0056 (W)	0.0398	3.63	Up
**34**	**Benzyl Alcohol**	38,134 (21,370)	10,457 (16,517)	0.0078 (W)	0.0449	3.65	Up
**35**	**3-Ureidopropionate**	38,579 (35,694)	9,876 (8,347)	0.0078 (W)	0.0449	3.91	Up
**36**	**Xylitol**	179,151 (149,300)	62,841 (46,556)	0.0078 (W)	0.0449	2.85	Up
**37**	**Uracil**	11,816 (3,281)	7,107 (3,562)	0.0188 (W)	0.0769	1.66	Up
**38**	**2-Amino-2-Methylpropanoate**	10,015 (6,279)	3,571 (5,177)	0.0188 (W)	0.0769	2.8	Up
**39**	**Alpha-Hydroxybutyric Acid**	9,227,011 (5,440,421)	3,461,640 (4,531,126)	0.0188 (W)	0.0769	2.67	Up
**40**	**Glycolaldehyde Dimer**	297,205 (203,567)	98,441 (177,487)	0.0244 (W)	0.0862	3.02	Up
**41**	**O-Acetyl-L-Serine**	281,630 (123,540)	146,712 (103,384)	0.0244 (W)	0.0862	1.92	Up
**42**	**Sarcosine**	1,101,826 (889,434)	299,357 (282,133)	0.0315 (W)	0.0885	3.68	Up
**43**	**N-Formylglycine**	13,264 (8,829)	7,172 (5,844)	0.0315 (W)	0.0885	1.85	Up
**44**	**N-Acetyl-L-Aspartic Acid**	17,571 (11,509)	9,907 (4,505)	0.0315 (W)	0.0885	1.77	Up
**45**	**L-Cystathionine**	28,881 (33,783)	5,004 (4,774)	0.0315 (W)	0.0885	5.77	Up
**46**	**Kynurenic Acid**	44,724 (33,962)	19,831 (26,431)	0.0315 (W)	0.0885	2.26	Up
**47**	**3-Hydroxy-3-Methylglutarate**	49,610 (23,936)	64,609 (22,885)	0.0315 (W)	0.0885	−1.3	Down
**48**	**3-Hydroxyanthranilate**	11,692 (18,306)	4,361 (7,424)	0.0400 (W)	0.108	2.68	Up

Table S4. ANOVA analysis of metabolites for high, medium and low doses of MSC-treated patients indicated 59 metabolites significantly changed among these groups, with 37 showing FDR differences of < 0.05 between cohorts.

	Metabolites	f. value	*p-value*	FDR	Fisher's LSD
1	Formyl-L-Methionyl Peptide	12.805	<0.0001	0.007	Low dose - Medium dose; Low dose - High dose; Medium dose - High dose
2	O-Acetyl-L-Serine	12.198	<0.0001	0.007	Low dose - Medium dose; Low dose - High dose; Medium dose - High dose
3	Methyl Vanillate	10.640	<0.0001	0.008	Low dose - Medium dose; Low dose - High dose; Medium dose - High dose
4	6-Carboxyhexanoate	10.607	<0.0001	0.008	Low dose - Medium dose; Low dose - High dose; Medium dose - High dose
5	N-Acetyl-L-Alanine	9.777	0.001	0.011	Low dose - Medium dose; Low dose - High dose; Medium dose - High dose
7	Urate	8.787	0.001	0.013	Low dose -Medium dose; Low dose - High dose; Medium dose - High dose
8	N-Acetyl-D-Mannosamine	8.309	0.002	0.013	Low dose - Medium dose; Low dose - High dose; Medium dose - High dose
9	Indole-3-Acetamide	8.305	0.002	0.013	Low dose - Medium dose; Low dose - High dose; Medium dose - High dose
10	3-Hydroxyanthranilate	8.231	0.002	0.013	Low dose - Medium dose; Low dose - High dose; Medium dose - High dose
11	Azelaic Acid	8.034	0.002	0.013	Low dose - Medium dose; Low dose - High dose; Medium dose - High dose
12	Hippurate	7.696	0.002	0.014	Low dose - Medium dose; Low dose - High dose; Medium dose - High dose
13	4-Acetamidobutanoate	7.688	0.002	0.014	Low dose - Medium dose; Low dose - High dose; Medium dose - High dose
14	Allantoin	7.156	0.003	0.018	Low dose - Medium dose; Low dose - High dose; Medium dose - High dose
15	Creatinine	6.828	0.004	0.020	Low dose - Medium dose; Low dose - High dose; Medium dose - High dose
16	4-Methyl-2-Oxo-Pentanoic Acid	6.768	0.004	0.020	Medium dose - Low dose; Low dose - High dose; Medium dose - High dose
17	N-Acetyl-L-Aspartic Acid	5.994	0.007	0.032	Low dose - Medium dose; Low dose - High dose; Medium dose - High dose

18	L-Tyrosine	5.904	0.008	0.032	Low dose -Medium dose; Low dose - High dose; Medium dose - High dose
19	L-Tryptophan	5.754	0.009	0.033	Low dose - Medium dose; Low dose - High dose; Medium dose - High dose
20	Pyruvic Aldehyde	5.479	0.011	0.038	Medium dose - Low dose; Low dose - High dose; Medium dose - High dose
21	N-Alpha-Acetyl-L-Lysine	5.022	0.015	0.050	Low dose - Medium dose; Low dose - High dose; Medium dose - High dose
22	Mannose	4.914	0.016	0.052	Medium dose - Low dose; Low dose - High dose; Medium dose - High dose
23	N-Amidino-L-Aspartate	4.806	0.017	0.052	Low dose - Medium dose; Low dose - High dose; Medium dose - High dose
24	Xanthosine	4.779	0.017	0.052	Low dose - Medium dose; Low dose - High dose; Medium dose - High dose
25	Propionate	4.717	0.018	0.053	Low dose - Medium dose; Low dose - High dose; Medium dose - High dose
26	Mono-Methyl Glutarate	4.649	0.019	0.053	Low dose - Medium dose; Low dose - High dose; Medium dose - High dose
27	Acetoin	4.548	0.021	0.055	Low dose - Medium dose; Low dose - High dose; Medium dose - High dose
28	L-Histidine	4.389	0.023	0.056	Medium dose - Low dose; Low dose - High dose; Medium dose - High dose
29	4-Pyridoxate	4.386	0.023	0.056	Low dose - Medium dose; Low dose - High dose; Medium dose - High dose
30	5-Hydroxy-L-Tryptophan	4.341	0.024	0.056	Low dose - Medium dose; Low dose - High dose; Medium dose - High dose
31	Suberic Acid	4.304	0.025	0.056	Low dose - Medium dose; Low dose - High dose; High dose - Medium dose
32	3-4-Hydroxyphenylpyruvate	4.289	0.025	0.056	Low dose - Medium dose; Low dose - High dose; Medium dose - High dose
33	Indole-3-Acetic Acid	4.158	0.028	0.060	Low dose - Medium dose; Low dose - High dose; Medium dose - High dose

34	Dl-5-Hydroxylysine	4.034	0.030	0.064	Low dose - Medium dose; Low dose - High dose; Medium dose - High dose
35	Guanosine	3.967	0.032	0.066	Low dose - Medium dose; Low dose - High dose
36	Hypotaurine	3.827	0.035	0.069	Low dose - Medium dose; Low dose - High dose; High dose - Medium dose
37	3-Ureidopropionate	3.810	0.036	0.069	Low dose - Medium dose; Low dose - High dose
38	Uridine-5-Monophosphate	3.769	0.037	0.069	Medium dose - Low dose; Low dose - High dose; Medium dose - High dose
39	Homogentisate	3.769	0.037	0.069	Low dose - Medium dose; Low dose - High dose; High dose - Medium dose
40	ADENINE	3.620	0.042	0.075	Low dose - Medium dose; Low dose - High dose; Medium dose - High dose
41	D---ARABINOSE	3.594	0.042	0.075	Low dose - Medium dose; Low dose - High dose; Medium dose - High dose
42	XYLITOL	3.449	0.048	0.077	Low dose - Medium dose; Low dose - High dose; Medium dose - High dose
43	D-PANTOTHENIC ACID	3.419	0.049	0.077	Medium dose - Low dose; Low dose - High dose; Medium dose - High dose

Table S5. HILIC-MS data showing MSC-treated septic shock patients compared to non-MSC-treated septic shock patients at 24 h. There are 8 metabolites with significant differences, but they do not have a significant FDR difference at 24 h post-MSC treatment.

Name	Mean (SD) of int. 24 h	Mean (SD) of obs. 24 h	*p*-value	*q*-value (FDR)	Fold change	Intervention/O Observation
**Diacetyl**	9,137 (2,558)	5,039 (2,808)	0.0052	0.3335	1.81	Up
**Uridine-5-Monophosphate**	2,708 (1,535)	972 (517)	0.0095	0.4094	2.78	Up
**Omega-Hydroxydodecanoic Acid**	12,750 (5,465)	7,045 (4,818)	0.032	0.6817	1.81	Up
**2-4-Dihydroxypteridine**	14,863 (1,573)	11,785 (3,618)	0.0393	0.6817	1.26	Up
**Melibiose**	25,543 (25,584)	4,981 (3,759)	0.0429	0.6817	5.13	Up
**3-Hydroxy-3-Methylglutarate**	39,560 (17,949)	59,277 (14,656)	0.0040 (W)	0.3335	−1.5	Down
**4-Hydroxybenzaldehyde**	90,172 (44,411)	55,535 (39,458)	0.0503 (W)	0.6817	1.62	Up

Table S6. Pathway analysis shows upregulation and downregulation of biochemical pathways in MSC-treated septic shock patients when compared to non-MSC-treated septic shock control patients using the most differentiating metabolites of HILIC-MS and NMR data.

Pathway	Total	Expected	Hits	Raw p	FDR	Impact	Change
Tryptophan metabolism	79	0.754	3	0.037	0.927	0.082	Upregulated
Argnine and Proline metabolism	77	0.799	3	0.043	0.388	0.164	Downregulated
Lysine metabolism	32	0.332	3	0.004	0.161	0.121	Downregulated
Citrate cycle (TCA cycle)	20	0.207	2	0.017	0.28	0.1	Downregulated
Aminoacyl-tRNA biosynthesis	75	0.778	4	0.006	0.178	0.056	Downregulated
Glycine, serine, and threonine metabolism	48	0.498	3	0.012	0.254	0.05	Downregulated
